# Towards new NIR dyes for free radical photopolymerization processes

**DOI:** 10.3762/bjoc.17.133

**Published:** 2021-08-16

**Authors:** Haifaa Mokbel, Guillaume Noirbent, Didier Gigmes, Frédéric Dumur, Jacques Lalevée

**Affiliations:** 1 Université de Haute-Alsace, CNRS, IS2M UMR 7361, F-68100 Mulhouse, France; 2 Université de Strasbourg, France; 3 Aix Marseille Univ, CNRS, ICR UMR 7273, F-13397 Marseille, France

**Keywords:** cyanine, NIR light, photochemistry

## Abstract

The use of cheap and safe near-infrared (NIR) light is still the subject of intense research efforts but remains a huge challenge due to the associated low photon energy (wavelength from 0.78 to 2.5 µm). In this study, a series of 17 NIR dyes mainly based on a well-established cyanine scaffold is proposed. Remarkably, 11 of them were never synthesized before. Markedly, noncharged structures, negatively charged cyanine bearing Na^+^ as counter cation, and positively charged cyanines bearing (B(Ph)_4_^−^) or (I^−^) as counter anions were examined as promising NIR light photoinitiating systems. Excellent photoinitiating abilities were found for some reported dyes when used in combination with iodonium salt and amine. Markedly, photothermal effects with a huge heater behavior were also observed for different NIR dye structures. Interestingly, the synthesis of interpenetrating polymer networks (IPNs, e.g., for the polymerization of acrylate/epoxy monomer blends) can also be carried out upon NIR light with the proposed systems.

## Introduction

Photopolymerization processes are well established due to the specific features and advantages. Indeed, the reaction is carried out at room temperature and with a high spatial resolution (the reaction only occurs in the light-irradiated areas). These latter photochemical processes are also very fast and efficient. However, energetic and unsafe UV light is usually used to ensure high polymerization rates and final reactive function conversions. To prevent the use of harmful UV light, different longer-wavelength photoinitiating systems (PISs) were proposed, e.g., blue light in dental materials [1–2]. Many PISs were developed for visible light, but much less studies are focused on the polymerization using near-Infrared (NIR) sources [3–8]. In this context, the use of NIR light is very attractive, i.e., such a long wavelength is quite safe and characterized by an excellent penetration in materials. Interestingly, NIR curing technology exhibit several advantages: i) an efficient process: a high polymerization rate obtained with low energy and a moderate device cost contrary to thermal solutions; ii) mild irradiation conditions: safer and cheaper systems; and iii) the curing of thick and/or filled systems that cannot be addressed with UV or even visible-light-sensitive systems. Thus, the NIR curing of thick and filled samples can be potentially used for composite materials and/or adhesives.

Both photochemical and photothermal NIR approaches were proposed to initiate polymerization, e.g., for free-radical processes, interpenetrating polymer network (IPN) synthesis, or photopolyaddition reactions [3–4,6–9]. NIR dyes, and more especially cyanine dyes, have been studied in NIR photosensitive systems [5–7,9–15]. The cyanine acts as a photosensitizer: it absorbs the light emitted in the NIR range and then acts with a combination of additives (oxidant agents and reducing agents) to generate initiating radicals.

**Scheme 1:**
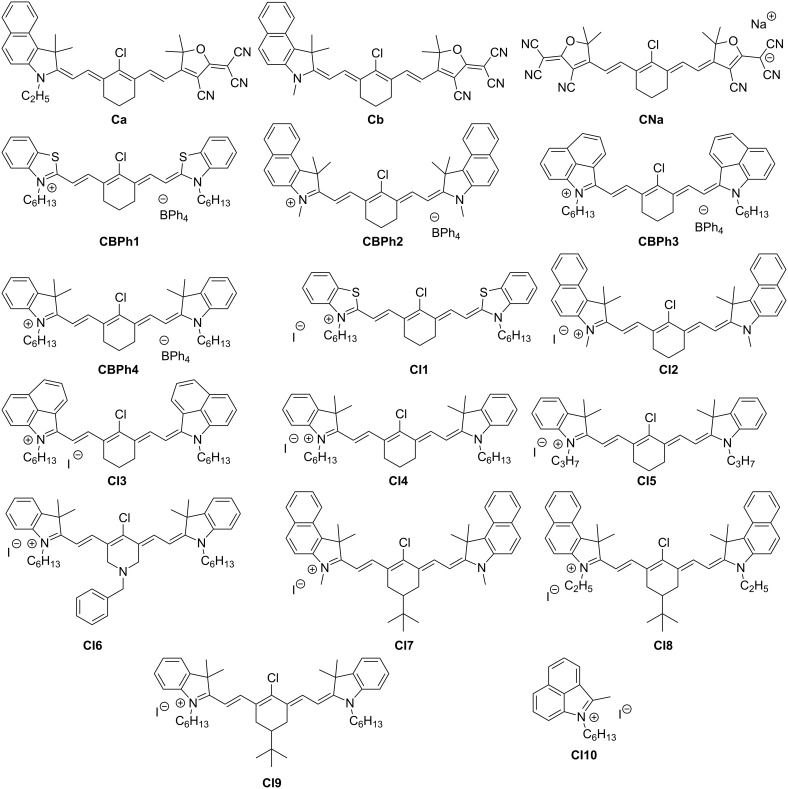
Investigated NIR dyes.

**Scheme 2:**
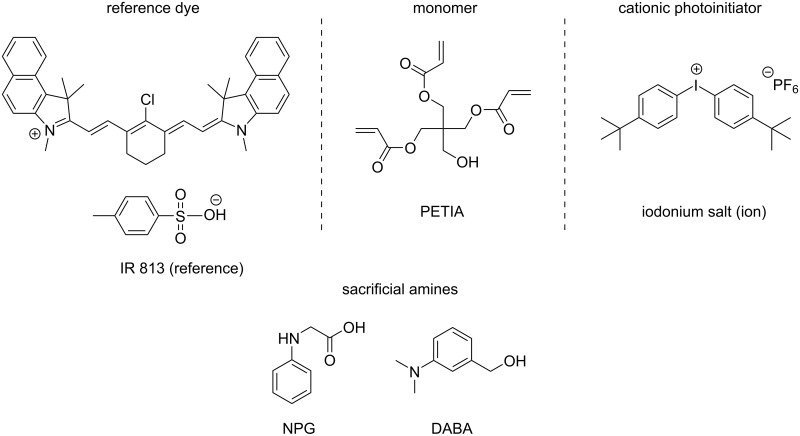
Other used chemicals.

In this work, a large series of 17 NIR dyes mainly based on a well-established cyanine scaffold is proposed (Scheme 1). Markedly, eleven of them were never synthesized before. These NIR dyes are studied in three-component systems in combination with an iodonium salt and the tertiary aromatic amines 3-(dimethylamino)benzyl alcohol (DABA) and *N*-phenylglycine (NPG), respectively, for the polymerization of a benchmark monomer (PETIA, Scheme 2). The polymerization profiles were recorded by real-time Fourier transform infrared spectroscopy (FTIR, the procedure is described in detail in [16]). Interestingly, a high polymerization-initiating ability was found with different dyes as well as photothermal effects with heater behaviors where the NIR dye acts as a light-to-heat convertor and transferred enough heat to the system to initiate the polymerization. The effects of counter anions and counter cations as well as cyanine structures are discussed.

## Results and Discussion

### Synthesis of the different dyes

**Scheme 3:**
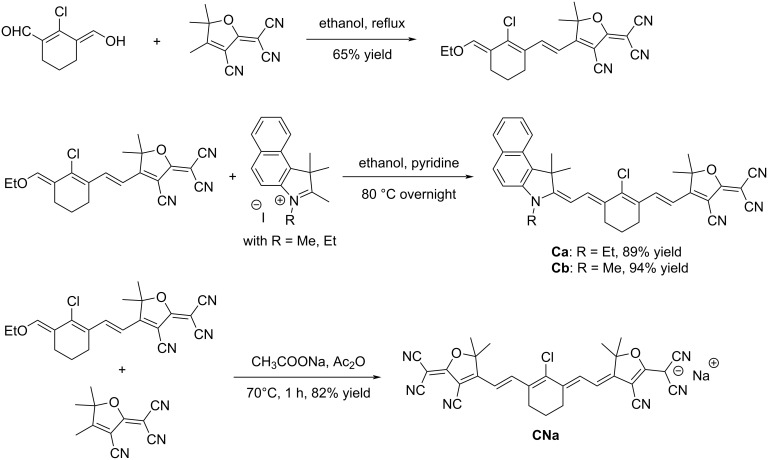
Synthetic routes to compounds **Ca**, **Cb**, and **CNa**.

Two different families of cyanines were prepared, differing by the way how the structure is formed. Notably, dyes **Ca** and **Cb** differ from the other cyanines by the asymmetric substitution. Thus, in the first step, the Claisen–Schmidt condensation of 2-chloro-3-(hydroxymethylene)cyclohex-1-ene-1-carbaldehyde on 2-(3-cyano-4,5,5-trimethylfuran-2(5*H*)-ylidene)malononitrile (TCF) furnished the intermediate 2-(4-((*E*)-2-((*E*)-2-chloro-3-(ethoxymethylene)cyclohex-1-en-1-yl)vinyl)-3-cyano-5,5-dimethylfuran-2(5*H*)-ylidene)malononitrile in 65% yield. A second Claisen–Schmidt condensation of 2-(4-((*E*)-2-((*E*)-2-chloro-3-(ethoxymethylene)cyclohex-1-en-1-yl)vinyl)-3-cyano-5,5-dimethylfuran-2(5*H*)-ylidene)malononitrile with the appropriate 3-alkyl-1,1,2-trimethyl-1*H*-benzo[*e*]indol-3-ium salt under basic conditions could furnish the two dyes **Ca** and **Cb** in 89% and 94% yield, respectively. Conversely, the anionic TCF-based heptamethine **CNa** was prepared under slightly different conditions, using sodium acetate in acetic anhydride. After one hour of reaction, compound **CNa** could be isolated in 82% yield (Scheme 3).

**Scheme 4:**
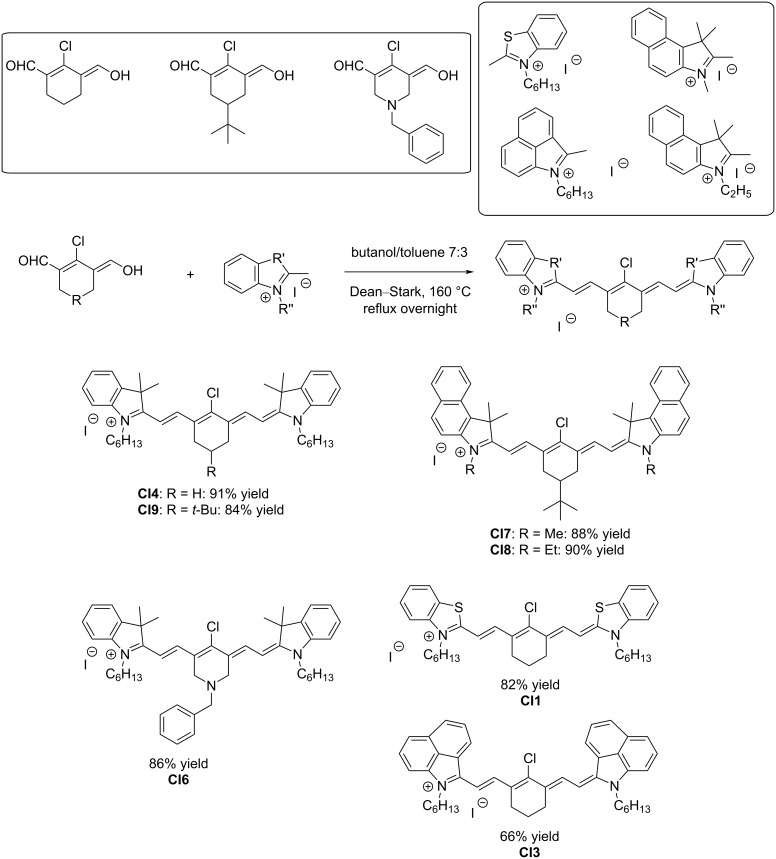
Synthetic routes to **CI1**, **CI3**, **CI4**, and **CI6**–**CI9**.

Due to the symmetrical substitution of the other dyes, a one-step procedure could be used for the synthesis of the pentamethine cyanine dyes. It has to be noticed that the present series of pentamethine dyes was prepared using synthetic protocols inspired by the literature. However, when dyes with already existing substitutions were reproduced in this work, original substitutions are also described, as exemplified with compound **CI6**. Indeed, for this compound, use of a central part derived from 1-benzylpiperidin-4-one is unprecedented in the literature. Similarly, among the seven symmetrical pentamethine dyes synthesized in this work, only dyes **CI8** [17] and **CI9** [18] were previously reported in the literature. For all the other dyes, judicious choice of the alkyl chains introduced to the peripheral groups resulted in previously unknown compounds. All dyes could be obtained with a reaction yield ranging from 66% to 91% (Scheme 4).

**Scheme 5:**
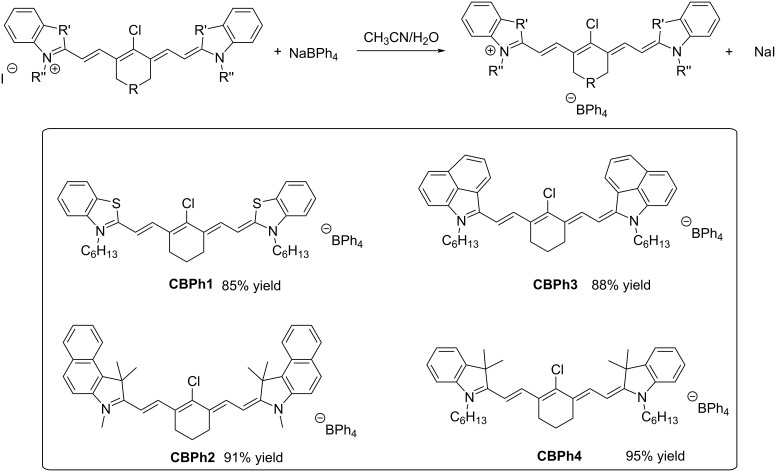
The metathesis reaction enabling the formation of “soft” salts **CBPh1-CBPh4**.

Finally, for some of the dyes, a metathesis reaction was carried out with sodium tetraphenylborate. Indeed, all dyes were obtained as iodine salts so that a counterion exchange could be carried out. Using this strategy, four “soft” salts could be obtained with a reaction yield ranging from 85% to 95% (see Scheme 5). It has to be noticed that this strategy has previously been reported in the literature [17–18].

### Light absorption properties of the different NIR dyes

**Figure 1:**
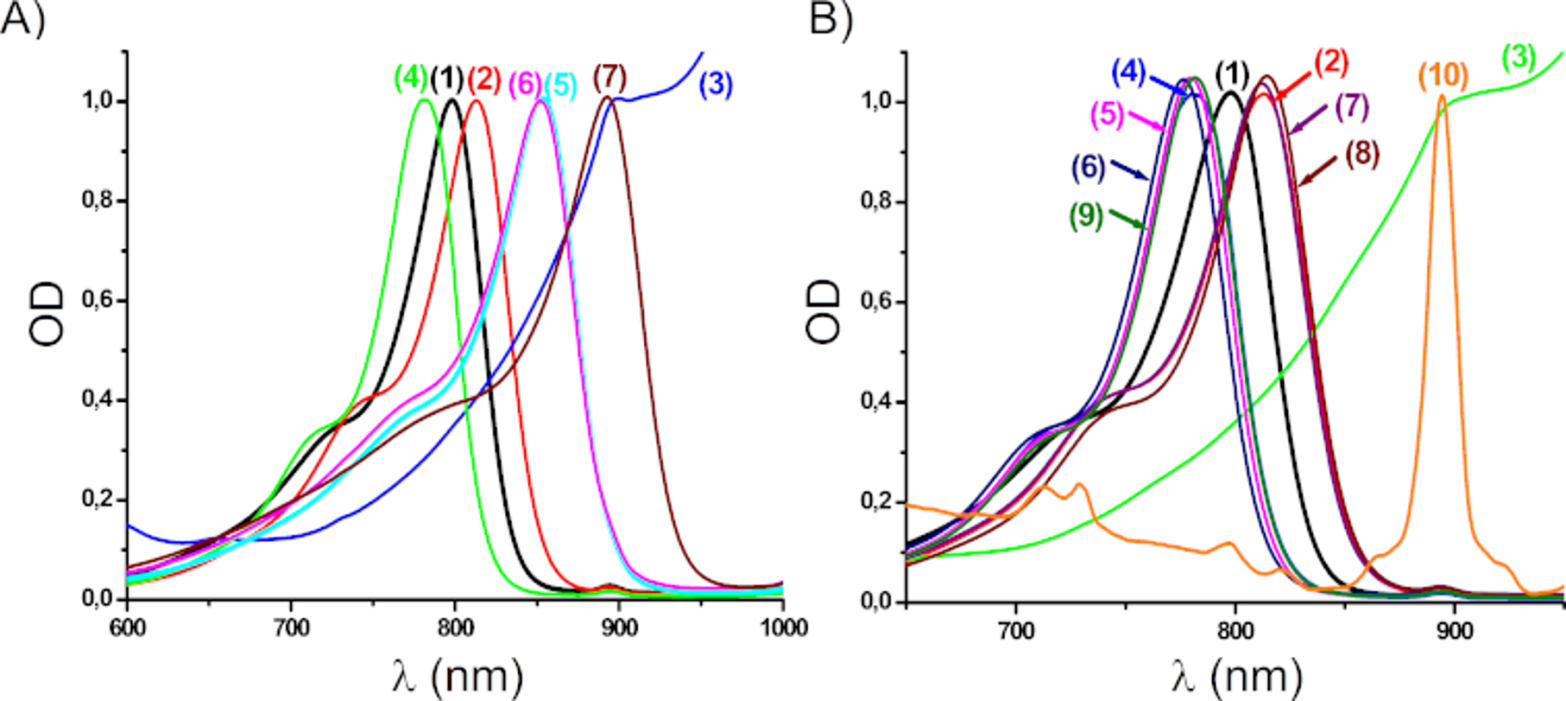
Visible–NIR spectra of NIR dyes in ACN. A) (1) **CBPh1**, (2) **CBPh2**, (3) **CBPh3**, (4) **CBPh4**, (5) **Ca**, (6) **Cb**, and (7) **CNa**. B) (1) **CI1**, (2) **CI2**, (3) **CI3**, (4) **CI4**, (5) **CI5**, (6) **CI6**, (7) **CI7**, (8) **CI8**, (9) **CI9**, and (10) **CI10**.

The light absorption properties of the different proposed dyes are depicted in Figure 1. They are characterized by a sharp and intense absorption band in the NIR range. Remarkably, absorption of most of the dyes is excellent in the 700–850 nm spectral range, ensuring fairly good overlap with the emission spectrum of the laser diode used in this work (i.e., LD@785 nm). Interestingly, no effect of the counter anion was observed (some of the dyes with two different counter ions were prepared and compared). In each case, we observed that the maximum absorption wavelength depends on the structure of the dye and not of the counter anion: i) λ_max_ = 798 nm for **CBPh1** and **CI1**; ii) λ_max_ = 810 nm for **CBPh2** and **CI2**; iii) λ_max_ = 900 nm for **CBPh3** and **CI3**; and iv) λ_max_ = 780 nm for **CBPh4** and **CI4**. However, the peripheral substituents in the cyanine scaffold play an important role in the absorption properties of some dyes, i.e., for **CBPh3**, **CI3**, and **CI10** bearing a 1-hexyl-2-methylbenzo[*cd*]indol-1-ium unit: a bathochromic shift of ≈100 nm was found for these two dyes, as compared to the other dyes investigated in this work, absorbing up to 850 nm. The latter, **CBPh3**, **CI3**, and **CI10**, exhibit a strong absorption above 850 nm (900–1000 nm) and a relatively low absorption at 785 nm (wavelength of the irradiation used). The visible–NIR spectra of IR 813 (Scheme 2) considered as a reference is given in Figure S1 in Supporting Information File 1.

### NIR polymerization initiating ability

Due to the good NIR absorption properties, the proposed dyes have been tested as photoinitiators in combination with an amine and an iodonium salt, iod, for the free radical polymerization of a benchmark acrylate monomer and compared to a reference initiating system based on IR 813 (Scheme 2) [7]. As the different dyes presented above exhibit good absorption properties at 785 nm, the photoinitiating abilities of the three-component systems (NIR dye/iod/amine) based on these dyes were further investigated upon irradiation with a LD@785 nm.

**Figure 2:**
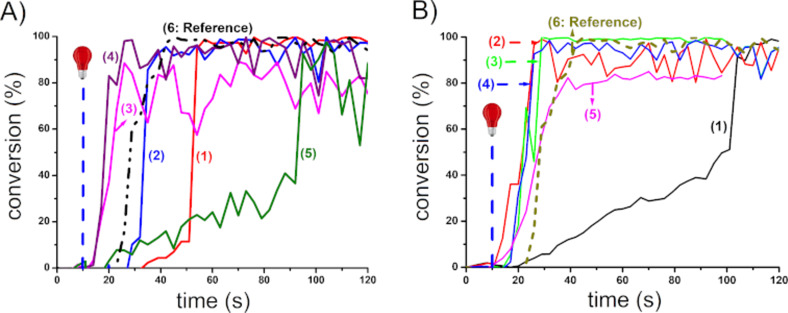
Photopolymerization profiles of PETIA monomer under air (acrylate functions conversion vs irradiation time) in the presence of a NIR dye/iod/NPG 0.1:3:2. %w/w/w system. A) NIR dyes with BPh_4_^−^ and Na^+^ as counter anion and counter cation: (1) **Ca**, (2) **Cb**, (3) **CBPh1**, (4) **CBPh4**, (5) **CNa**, and (6) IR 813. B) NIR dyes with I^−^ as counter anion: (1) **CI3**, (2) **CI4**, (3) **CI5**, (4) **CI8**, (5) **CI9**, and (6) IR 813; upon exposure to a 785 nm laser diode (0.9 W/cm^2^), thickness = 1.4 mm. The irradiation starts at *t* = 10 s.

**Figure 3:**
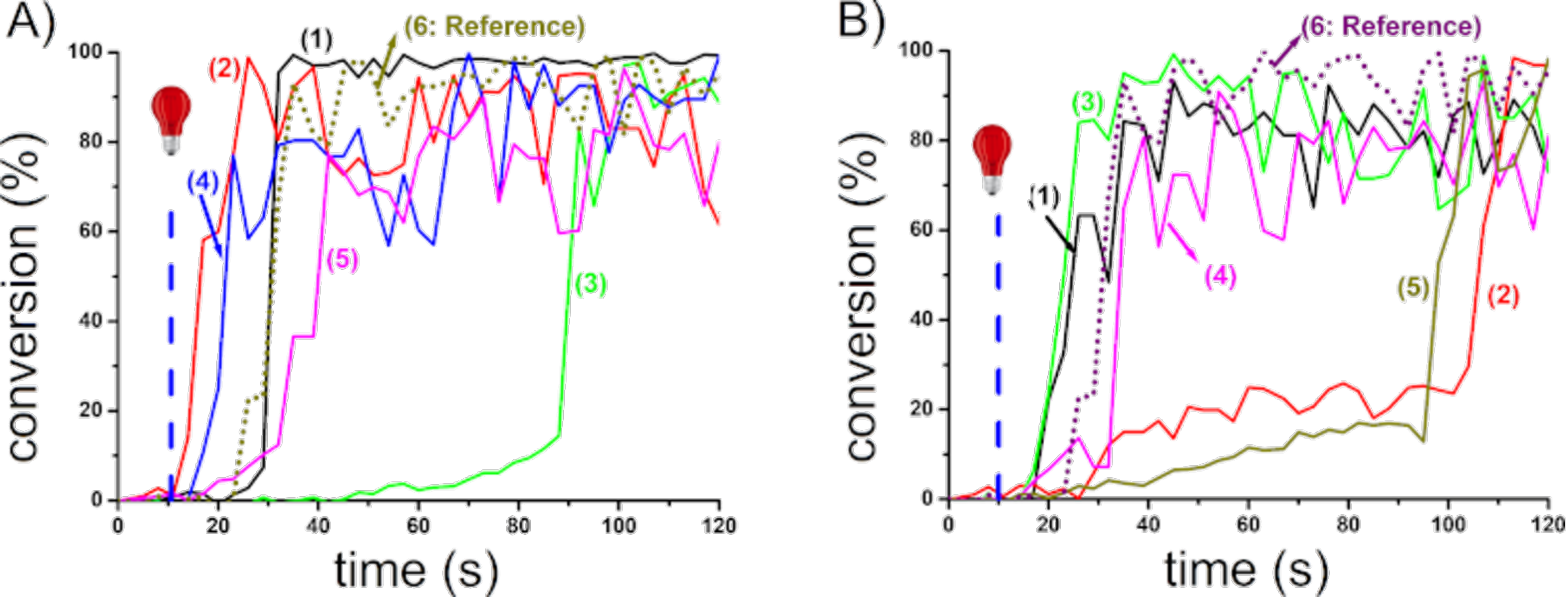
Photopolymerization profiles of PETIA monomer under air (acrylate functions conversion vs irradiation time) in the presence of a NIR dye/iod/DABA 0.1:3:2, %w/w/w system. A) NIR dyes with BPh_4_^−^ and Na^+^ as counter anion and counter cation: (1) **Cb**, (2) **CBPh1**, (3) **CBPh3**, (4) **CBPh4**, (5) **CNa**, and (6) IR 813. B) NIR dyes with I^−^ as counter anion: (1) **CI1**, (2) **CI3**, (3) **CI5**, (4) **CI9**, (5) **CI10**, and (6) IR 813; upon exposure to a 785 nm laser diode (0.9 W/cm^2^), thickness = 1.4 mm. The irradiation starts at *t* = 10 s.

**Scheme 6:**
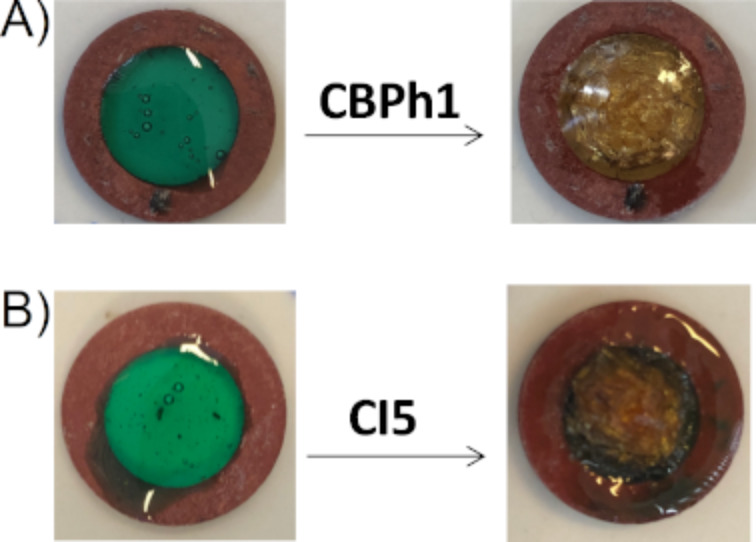
Pictures of polymers obtained for a thickness of 1.4 mm, using a NIR dye/iod/amine 0.1:3:2, %w/w/w system. A) amine = NPG and B) amine = DABA; upon exposure to a 785 nm laser diode (0.9 W/cm^2^) for 120 seconds under air.

Using DABA instead of NPG as the amine in the proposed three-component systems, the resulting polymerization profiles obtained with NPG and DABA, respectively, are depicted in Figure 2 and Figure 3. The NIR dyes proposed have strong abilities to initiate the free radical polymerization. Indeed, the polymers obtained are tack-free after only a few seconds of irradiation (Scheme 6). Without NIR dyes, no polymerization occurs. At 785 nm, in most cases, the final acrylate function conversion (FC) measured were higher than 80%. Such a high reactive function conversion is quite high for multifunctional monomers (with PETIA being a trifunctional acrylic monomer). Moreover, for the majority of the proposed systems, the photopolymerization starts as soon as the light is turned on. This can be explained by a low oxygen inhibition, which is an advantage for different applications where the photopolymerization under air is required. As shown in Figure 2 and Figure 3, only **CNa**, **CBPh3**, **CI3**, and **CI10** show a long inhibition time, and this can be ascribed to the low absorption of these dyes at the wavelength of the irradiation used here. However, despite the low absorptions at 785 nm, the photopolymerization is still successful.

Interestingly, no major difference was observed between the two amines used (NPG as shown in Figure 2 vs DABA as shown in Figure 3). However, in most cases, while using DABA as the base, slightly higher final conversion could be reached, and the process was also more rapid (Table 1). Therefore, the amine structure has an influence on the polymerization process: the interaction existing between the dye and the amine is an important parameter governing the formation of initiating radicals. The slightly lower reactivity of NPG vs DABA can probably be ascribed to a lower production of initiating radicals in this case, rendering the associated PIS slightly less efficient to overcome the oxygen inhibition. This behavior has already been observed in other works when other NIR dyes were used in three-component PISs comprising an oxidant agent and an amine [8]. In all cases, NIR dyes proposed showed excellent reactivity using different amines and an iodonium salt. This suggests that an NIR approach is an elegant way for fast curing processes upon mild irradiation conditions (much longer wavelength than UV light).

**Table 1:** Quantitative estimations of the polymerization time of PETIA monomer using a NIR dye/iod/amine 0.1:3:2, w/w/w system upon exposure to a laser diode at 785 nm (0.9 W/cm²), thickness = 1.4 mm, under air and at room temperature. The irradiation starts at *t* = 10 s.

category	compound	NPG		DABA	
		curing time^a^	exothermicity^b^	curing time	exothermicity

no counter ion	**Ca**	97% in 55 s	++	40% in >120 s	+
	**Cb**	96% in 41 s	++	97% in 35 s	++
Na^+^ as counter cation	**CNa**	90% in 95 s	++	80% in 42 s	+
borate as counter anion	**CBPh1**	87% in 26 s	+	92% in 18 s	+
	**CBPh2**	—^c^	-	90% in 26 s	++
	**CBPh3**	50% in >120 s	+	95% in 100 s	+
	**CBPh4**	97% in 27 s	++	89% in 36 s	++
I^−^ as counter anion	**CI1**	40% in >120 s	+	84% in 35 s	+
	**CI2**	—^c^	-	92% in 34 s	+
	**CI3**	95% in 110 s	++	97% in 113 s	++
	**CI4**	99% in 26 s	+	60% in >120 s	+
	**CI5**	99% in 29 s	++	95% in 34 s	+
	**CI6**	90% in 34 s	+	90% in 50 s	++
	**CI7**	40% >120 s	+	40% in >120 s	+
	**CI8**	95% in 26 s	++	84% in 45 s	+
	**CI9**	94% in 32 s	+	90% in 45 s	+
	**CI10**	10% in >120 s	+	92% in 105 s	++
reference compound	IR 813	97% in 43 s	++	97% in 37 s	++

^a^Time required to reach high final conversion. ^b^+: steams; ++: steams and cracks. ^c^Not stable: polymerization upon stirring.

**Scheme 7:**
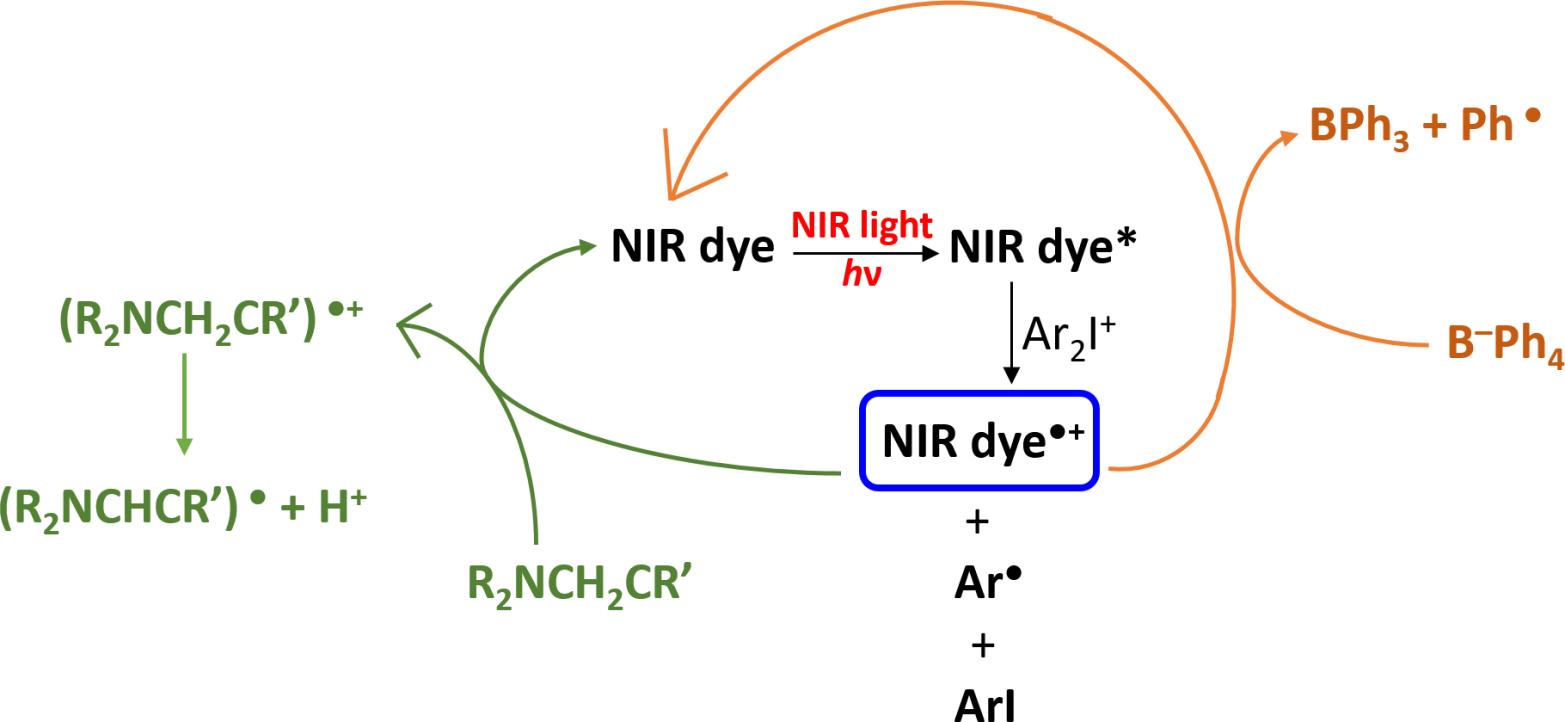
Proposed mechanism for the photochemical reactivity of NIR dyes in a three-component PIS.

As shown in the Scheme 1, the dyes investigated in this work differ by the counter anion and cation, respectively. By taking into account the effect of the counter ion, the trend in efficiency follows the order BPh_4_^–^ > I^–^ > Na^+^. The polymerization is much more efficient when borate is used as the counter anion with both a higher polymerization rate and FC. The results show that the formation of initiating radicals can be related to the decomposition of the borate moiety. This can be explained by the fact that the borate counter ion can act as an electron-donating component with the radical cation dye^•+^ and therefore, the dye/borate system is able to generate additional free radicals in the reaction medium, improving, in turn, the polymerization process (Scheme 7). This behavior has already been reported in the literature and is in full agreement with the proposed mechanism [4].

More particularly, it is interesting to compare the new proposed dye with IR 813, used as a benchmark structure. Under the same conditions, many dyes show a reactivity similar to IR 813. We noticed that IR 813 shows an excellent reactivity in NIR photopolymerization [7]. Remarkably, in this work, no inhibition time was observed while using different dyes, i.e., **CBPh1**, **CBPh4**; **CI1**, and **CI5**, as compared to IR 813. All these observations suggest that the proposed photoinitiating NIR dye/iod/amine compositions result in high-performance NIR-photosensitive systems.

Polymerization of an acrylate-based monomer causes the release of heat from each polymerizing double bond (−83.6 kJ/mol) [19]. This allows an exothermic reaction in different systems based on the reactivity but also due to the heater behavior of the NIR dye, i.e., a high light-to-heat (photothermal) conversion process is expected with different NIR dyes [9]. Table 1 and Figure S2 (Supporting Information File 1) clearly show the exothermicity observed upon irradiation.

**Figure 4:**
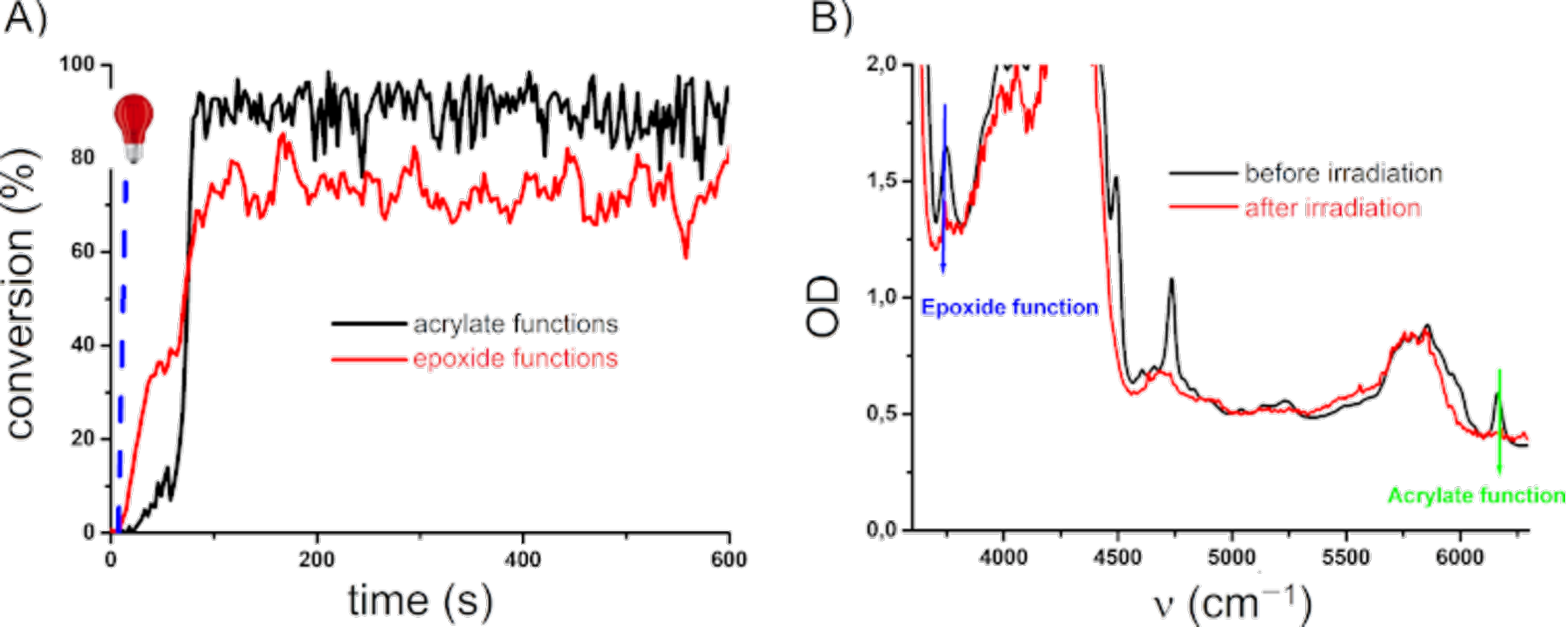
A) Photopolymerization profiles of PETIA/epoxy blend 1:1, w/w under air (acrylate and epoxy functions conversion vs irradiation time) in the presence of **Cb**/iod 0.3:2, w/w upon exposure to a laser diode at 785 nm (0.9 W/cm^2^), thickness = 1.4 mm. The irradiation starts at *t* = 10s. B) FTIR spectra before and after irradiation.

The proposed NIR dye/iodonium combinations are able to generate free radicals and cationic species (Scheme 7 and Figure 4), and these two-component systems can also be used for the preparation of IPNs through the polymerization of acrylate/epoxy monomer blends (3,4-epoxycyclohexylmethyl-3,4-epoxycyclohexane carboxylate from Allnex was used as benchmark epoxy monomer) [20]. The formation of IPNs can be highly worthwhile to reach better polymer mechanical properties or lower shrinkage than pure radical polymerization.

## Conclusion

In the present study, a number of NIR-absorbing dyes has been investigated as potential NIR PISs. The NIR curing of acrylate monomer is proposed in the presence of three-component PISs (NIR dye/iod/amine). The free radical polymerization upon exposure to the laser diode at 785 nm was successfully initiated using new synthesized dyes. This can be used to obtain tack-free polymers after only a few seconds of NIR irradiation. Markedly, IPNs can also be prepared with NIR light. Potential applications and investigations of the mechanical properties of the generated polymers and composites will be provided in the forthcoming works.

## Supporting Information

File 1 Additional figures and experimental data.
