# Supplementary material for: Towards new NIR dyes for free radical photopolymerization processes
Source: Beilstein J Org Chem. 2021 Aug 16;17:2067–76. doi: 10.3762/bjoc.17.133 (PMC8381812; doi:10.3762/bjoc.17.133)
Supplement: File 1 — Additional figures and experimental data. [file Beilstein_J_Org_Chem-17-2067-s001.pdf]

BEILSTEIN JOURNAL OF ORGANIC CHEMISTRY

Supporting Information

for

Towards new NIR dyes for free radical photopolymerization processes

Haifaa Mokbel, Guillaume Noirbent, Didier Gigmes, Frédéric Dumur and Jacques Lalevée

Beilstein J. Org. Chem. **2021,** *17,* 2067–2076. doi:10.3762/bjoc.17.133

Additional figures and experimental data

License and Terms: This is a supporting information file under the terms of the Creative Commons Attribution License (https://creativecommons.org/licenses/by/4.0). Please note that the reuse, redistribution and reproduction in particular requires that the author(s) and source are credited and that individual graphics may be subject to special legal provisions.
The license is subject to the *Beilstein Journal of Organic Chemistry* terms and conditions: (https://www.beilstein-journals.org/bjoc/terms)

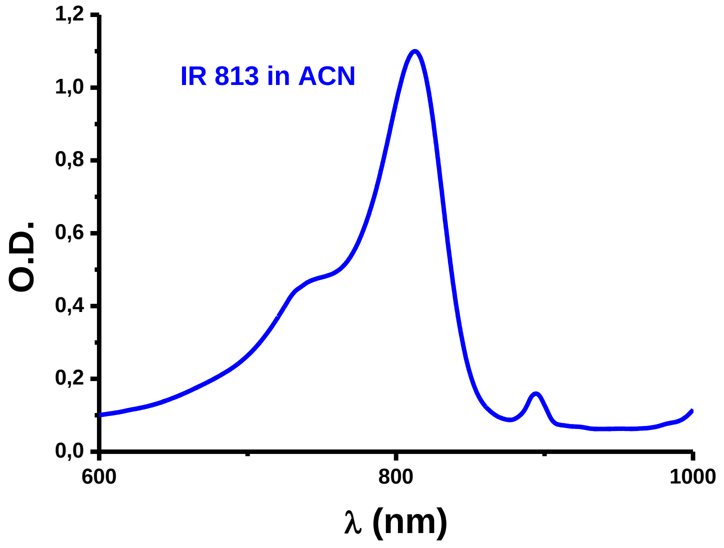

Figure S1: Visible–NIR spectrum of IR 813 in ACN.

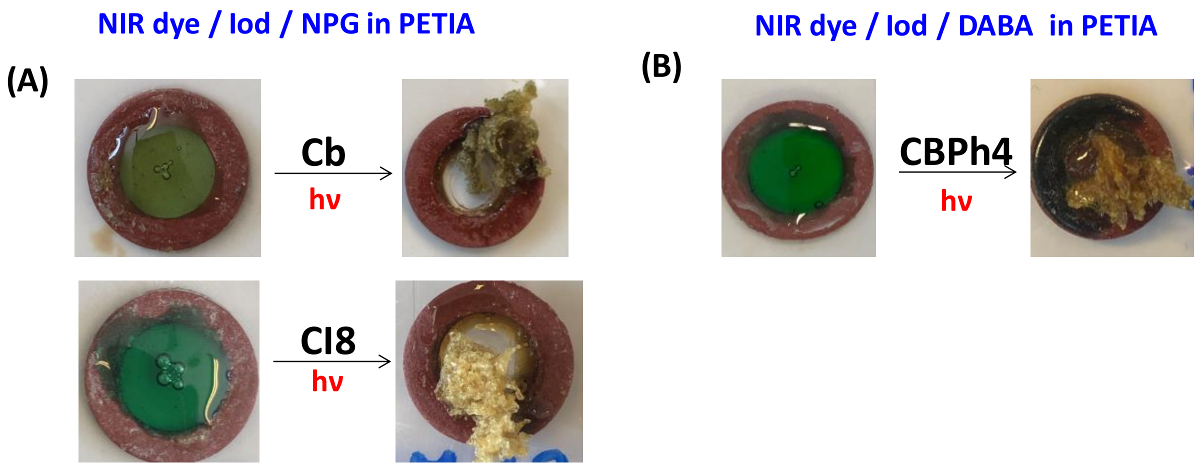

Figure S2: Picture of composites obtained for a thickness = 1.4 mm, using NIR dye/iod/amine 0.1:3:2, wt %. A) amine = NPG, B) amine = DABA; upon exposure to a laser diode at 785 nm (0. 9 W/cm^2).

Experimental section

Dye synthesis

All reagents and solvents were purchased from Aldrich or Alfa Aesar and used as received without further purification. Mass spectroscopy was performed by the Spectropole of Aix-Marseille University. ESI mass spectral analyses were recorded with a 3200 QTRAP (Applied Biosystems SCIEX) mass spectrometer. The HRMS analysis was performed with a QStar Elite (Applied Biosystems SCIEX) mass spectrometer. Elemental analyses were recorded with a Thermo Finnigan EA 1112 elemental analysis apparatus driven by the Eager 300 software. ^{1}H and ^{13}C NMR spectra were determined at room temperature in 5 mm o.d. tubes on a Bruker Avance 400 spectrometer of the Spectropole: ^{1}H (400 MHz) and ^{13}C (100 MHz). The ^{1}H chemical shifts were referenced to the solvent peaks: DMSO (2.49 ppm), $CDCl_3$ (7.26 ppm), and the ^{13}C chemical shifts were referenced to the solvent peaks: DMSO (49.5 ppm) and $CDCl_3$ (77.0 ppm), respectively. All photoinitiators were prepared with analytical purity up to accepted standards for new organic compounds (>98%), which were checked by high-field NMR analysis. **CI5** was purchased from Aldrich and used as received.

Synthesis of 2-chloro-3-(hydroxymethylene)cyclohex-1-ene-1-carbaldehyde

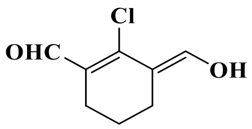

Chemical Formula: $C_8H_9ClO_2$
Molecular Weight: 172.6080

To a chilled solution of DMF (20 mL, 273 mmol, 5.4 equiv) in DCM (20 mL) was added dropwise at 0 °C a solution of $POCl_3$ (17.5 mL dissolved in 20 mL DCM). After 30 min, cyclohexanone (5 g, 50 mmol, 1 equiv) was added and the resulting mixture was refluxed with vigorous stirring for 3 h at 80 °C, poured into ice-cold water, and kept overnight to obtain the

title compound as a yellow solid (8.11 g, 94% yield). ^{1}H NMR (400 MHz, $CDCl_3$) δ 10.12 (s, 1H), 2.54 (s, 1H), 2.35 (t, J = 6.4 Hz, 4H), 1.57 (m, 2H). Analyses were consistent with those previously reported in the literature [1].

Synthesis of 5-(*tert*-butyl)-2-chloro-3-(hydroxymethylene)cyclohex-1-ene-1-carbaldehyde

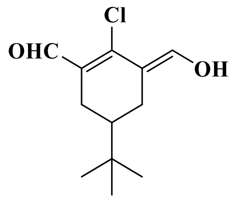

Chemical Formula: $C_{12}H_{17}ClO_2$
Molecular Weight: 228.7160

To a chilled solution of DMF (20 mL, 273 mmol, 5.4 equiv) in DCM (20 mL) was added dropwise at 0 °C a solution of $POCl_3$ (17.5 mL dissolved in 20 mL DCM). After 30 min, 4-*tert*-butylcyclohexanone (7.72 g, 50 mmol, 1 equiv, *M* = 154.25 g/mol) was added and the resulting mixture was refluxed with vigorous stirring for 3 h at 80 °C, poured into ice-cold water, and kept overnight to obtain the title compound as a yellow solid (10.86 g, 95% yield). Due to its low stability, this compound should be stored in the fridge at 2−4 °C. ^{1}H NMR (400 MHz, DMSO-d_6) δ 8.83 (s, 1H), 2.81 – 2.70 (m, 2H), 2.55 (s, 1H), 1.77 (dd, J = 15.5, 12.7 Hz, 2H), 1.22 (tt, J = 12.4, 3.8 Hz, 1H), 0.91 (s, 9H); HRMS (ESI MS) m/z: theor: 228.0917 found: 228.0920 ($M^{+\cdot}$ detected).

Synthesis of 1-benzyl-4-chloro-5-(hydroxymethylene)-1,2,5,6-tetrahydropyridine-3-carbaldehyde

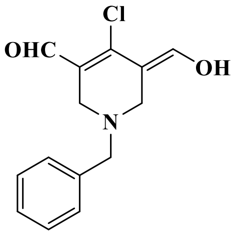

Chemical Formula: $C_{14}H_{14}ClNO_2$
Molecular Weight: 263.7210

To a chilled solution of DMF (20 mL, 273 mmol, 5.4 equiv) in DCM (20 mL) was added dropwise at 0 °C a solution of $POCl_3$ (17.5 mL dissolved in 20 mL DCM). After 30 min, 1-benzylpiperidin-4-one (9.46 g, 50 mmol, 1 equiv, *M* = 189.26 g/mol) was added and the resulting mixture was refluxed with vigorous stirring for 3 h at 80 °C, poured into ice-cold water, and kept overnight to obtain the title compound as a brown solid (12.13 g, 92% yield). Due to its low stability, this compound should be stored in the fridge at 2-4 °C. It was directly engaged in the next step.

Synthesis of 1,1,2,3-tetramethyl-1*H*-benzo[*e*]indol-3-ium iodide

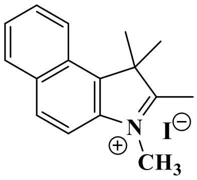

Chemical Formula: $C_{16}H_{18}IN$
Molecular Weight: 351,23

In a manner analogous to [2], the synthesis was carried out. 1,1,2-Trimethyl-1*H*-benz[e]indole (1.31 g, 6.23 mmol, 1 equiv, *M* = 209.12 g/mol) was dissolved in 5 mL 2-ethoxyethanol. Iodomethane (1.33 g, 9.34 mmol, 1.5 equiv, *M* = 141.94 g/mol) was added to the solution, and the mixture was heated at 110 °C for 16 h under an argon atmosphere. Then, the precipitate was collected by filtration, and washed with diethyl ether to afford the title compound as a gray

solid without further purification (2.06 g, 94% yield). ^{1}H NMR (400 MHz, DMSO-d_6) δ 8.36 (d, *J* = 8.5 Hz, 1H), 8.29 (d, *J* = 8.9 Hz, 1H), 8.22 (d, *J* = 7.8 Hz, 1H), 8.10 (d, *J* = 8.9 Hz, 1H), 7.79 (ddd, *J* = 8.4, 6.9, 1.4 Hz, 1H), 7.72 (ddd, *J* = 8.0, 6.9, 1.1 Hz, 1H), 4.09 (d, *J* = 0.6 Hz, 3H), 2.87 (d, *J* = 0.6 Hz, 3H), 1.75 (s, 6H); ^{13}C NMR (100 MHz, $CDCl_3$) δ 195.92; 142.07, 141.55, 129.28, 128.72, 123.34; 115.13, 53.96, 34.95, 34.93, 21.79, 14.42; HRMS (ESI MS) m/z: theor: 224.1434 found: 224.1435 (M^+ detected). Analyses are consistent with those previously reported in the literature [3].

Synthesis of 3-ethyl-1,1,2-trimethyl-1*H*-benzo[*e*]indol-3-ium iodide

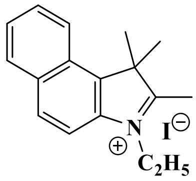

Chemical Formula: $C_{17}H_{20}IN$
Molecular Weight: 365.2585

1,1,2-Trimethyl-1*H*-benz[*e*]indole (1.31 g, 6.23 mmol, 1 equiv, *M* = 209.12 g/mol) was dissolved in 5 mL 2-ethoxyethanol. Iodoethane (1.46 g, 9.34 mmol, 1.5 equiv, *M* = 155.97 g/mol) was added to the solution, and the mixture was heated at 110 °C for 16 h under an argon atmosphere. Then, the precipitate was collected by filtration, and washed with diethyl ether to afford the title compound as a gray solid without further purification (2.16 g, 95% yield). ^{1}H NMR (400 MHz, DMSO-d_6) δ 8.38 (d, *J* = 8.4 Hz, 1H), 8.31 (d, *J* = 8.9 Hz, 1H), 8.23 (d, *J* = 7.8 Hz, 1H), 8.16 (d, *J* = 8.9 Hz, 1H), 7.80 (ddd, *J* = 8.4, 6.9, 1.4 Hz, 1H), 7.77 – 7.71 (m, 1H), 4.63 (q, *J* = 7.3 Hz, 2H), 2.95 (s, 3H), 1.77 (s, 6H), 1.52 (t, *J* = 7.3 Hz, 3H); ^{13}C NMR (100 MHz, DMSO-d_6) δ 195.91,138.24, 137.03, 133.08, 130.71, 129.73, 128.49, 127.33, 127.26, 123.41, 113.27, 55.55, 43.46, 21.53, 13.67, 12.93; HRMS (ESI MS) m/z: theor: 238.1590 found: 238.1590 ($M^{+\cdot}$ detected). Analyses are consistent with those previously reported in the literature [4].

Synthesis of 1-hexylbenzo[*cd*]indol-2(1*H*)-one

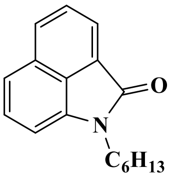

Chemical Formula: $C_{17}H_{19}NO$
Molecular Weight: 253.3450

NaH (0.6 g, 15 mmol, *M* = 24 g/mol, 60% dispersed in mineral oil) was added into a solution of benzo[*cd*]indol-2(1*H*)-one (846 mg, 5 mmol) in 15 mL of anhydrous DMF under Ar atmosphere. Then the mixture was cooled to 0 °C, and iodohexane (1.27 g, 0.88 mL, 6 mmol, *M* = 212.07 g/mol, ρ = 1.437 g/mL) was added dropwise. The mixture was stirred at room temperature for 3 h and then extracted with ethyl acetate, washed with brine, and concentrated. The crude product was then purified by column chromatography to give a yellow solid. ^1H NMR (400 MHz, $CDCl_3$) δ 8.05 (d, J = 7.0 Hz, 1H), 8.00 (d, J = 8.1 Hz, 1H), 7.70 (dd, J = 8.1, 7.0 Hz, 1H), 7.52 (d, J = 8.3 Hz, 1H), 7.46 (dd, J = 8.5, 6.9 Hz, 1H), 6.91 (d, J = 6.9 Hz, 1H), 3.91 (t, J = 7.3 Hz, 2H), 1.78 (dt, J = 15.1, 7.5 Hz, 2H), 1.47 – 1.25 (m, 11H), 0.87 (t, J = 7.1 Hz, 3H); Anal. calc. for $C_{17}H_{19}NO$: C, 80.6, H, 7.6, O, 6.3; found: C 80.4, H 7.8, O 6.4; HRMS (ESI MS) m/z: theor: 254.1539 found: 254.1540 ($[M+H]^+$ detected).

Synthesis of 3-hexyl-2-methylbenzo[*d*]thiazol-3-ium iodide

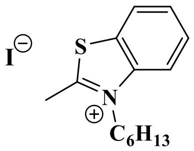

Chemical Formula: $C_{14}H_{20}INS$
Molecular Weight: 361.2855

2-Methylbenzo[*d*]thiazole (1 g, 6.7 mmol, *M* = 149.21 g/mol) and 1-iodohexane (7.1 g, 33 mmol, *M* = 212.07 g/mol) were heated overnight at 110 °C under nitrogen atmosphere. Upon cooling, the product was obtained as a gray solid that was washed with Et_2O and dried under high vacuum. It was isolated in quantitative yield. ^1H NMR (400 MHz, $CDCl_3$) δ : 8.33 (d, 1H,

J = 8.5 Hz), 7.99 (d, 1H, J = 8.5 Hz), 7.82 (td, 1H, J = 7.3 Hz, J = 0.9 Hz), 7.74 (td, 1H, J = 7.3 Hz, J = 0.9 Hz), 4.85 (t, 2H, J = 7.9 Hz), 3.45 (s, 3H), 1.96 (qt, 2H, J = 6.9 Hz), 1.50 (qt, 2H, J = 6.9 Hz), 1.53-1.29 (m, 4H), 0.89 (t, 3H, J = 6.9Hz); ^{1}H NMR (100 MHz, $CDCl_3$) δ : 175.1, 141.0, 130.0, 129.1, 128.7, 124.7, 116.5, 51.6, 31.2, 28.7, 26.5, 22.4, 19.9, 13.9; HRMS (ESI MS) m/z: theor: 361.0361 found: 361.0365 (M^+ detected).

Synthesis of 2-(4-((*E*)-2-((*E*)-2-chloro-3-(ethoxymethylene)cyclohex-1-en-1-yl)vinyl)-3-cyano-5,5-dimethylfuran-2(5*H*)-ylidene)malononitrile

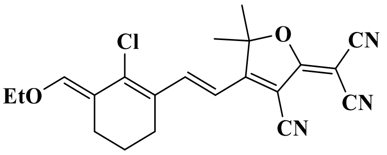

Chemical Formula: $C_{21}H_{20}ClN_3O_2$
Molecular Weight: 381.8600

2-Chloro-3-(hydroxymethylene)cyclohex-1-ene-1-carbaldehyde (0.5 g, 2.9 mmol, 1 equiv, *M* = 172.61 g/mol) and 2-(3-cyano-4,5,5-trimethylfuran-2(5*H*)-ylidene)malononitrile (0.58 g, 2.9 mmol, 2 equiv, *M* = 199.21 g/mol) were dissolved in ethanol (20 mL) and the solution was refluxed overnight. The solvent was removed under reduced pressure. The residue was filtered on a plug of silica gel (SiO_2) using DCM/acetone 9:1 as the eluent. After evaporation of the volatiles, dissolution in a minimum of DCM followed by the addition of pentane precipitated a purple solid that was filtered off, washed several times with pentane (3 times) and dried under vacuum (0.72 g, 65% yield). ^{1}H NMR (400 MHz, $CDCl_3$) δ 8.06 (d, *J* = 15.9 Hz, 1H), 7.20 (s, 1H), 6.47 (d, *J* = 15.9 Hz, 1H), 4.12 (q, *J* = 7.1 Hz, 2H), 2.55 – 2.46 (m, 4H), 1.85 – 1.71 (m, 8H), 1.37 (t, *J* = 7.1 Hz, 3H); ^{13}C NMR (100 MHz, $CDCl_3$) δ 175.81, 174.53, 153.84, 144.38, 142.98, 128.13, 116.43, 113.43, 112.36, 111.57, 110.73, 98.15, 97.42, 70.77, 56.65, 27.15, 26.53, 23.95, 20.68, 15.62; HRMS (ESI MS) m/z: theor: 382.1317 found: 382.1312 ($[M+H]^+$ detected). Analyses are consistent with those previously reported in the literature [5].

Synthesis of 2-(4-((*E*)-2-((*E*)-2-chloro-3-((*E*)-2-(3-ethyl-1,1-dimethyl-1,3-dihydro-2*H*-benzo[*e*]indol-2-ylidene)ethylidene)cyclohex-1-en-1-yl)vinyl)-3-cyano-5,5-dimethylfuran-2(5*H*)-ylidene) malononitrile (compound **Ca)**

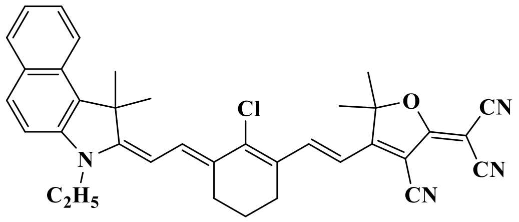

Chemical Formula: $C_{36}H_{33}ClN_4O$
Molecular Weight: 573.1370

2-(4-((*E*)-2-((*E*)-2-Chloro-3-(ethoxymethylene)cyclohex-1-en-1-yl)vinyl)-3-cyano-5,5-dimethylfuran-2(5*H*)-ylidene)malononitrile (0.5 g, 1.31 mmol, 1 equiv, *M* = 381.86 g/mol) and 3-ethyl-1,1,2-trimethyl-1*H*-benzo[*e*]indol-3-ium iodide (0.57 g, 1.57 mmol, 1.2 equiv, *M* = 365.26 g/mol) were dissolved in ethanol (50 mL), and anhydrous pyridine (0.16 g, 0.16 mL, 1.96 mmol, 1.5 equiv, *M* = 79.10 g/mol, ρ = 0.978) was added. The reaction mixture was stirred for 18 h at 80 °C. The solvent was evaporated under reduced pressure, the residue was dissolved in 20 mL of dichloromethane and washed with distilled water (2 × 20 mL). The organic layer was dried over anhydrous magnesium sulfate, filtered, and concentrated. The crude solid was purified by flash chromatography on silica gel (manual chromatography using air pressure to speed up the flow, silica gel high purity grade, pore size 60 Å, 230–400 mesh particle size, 40–63 µm particle size, for flash chromatography, Sigma Aldrich) using dichloromethane as eluent to afford the product as a green solid (668, 89% yield). ^{1}H NMR (400 MHz, DMSO-d_6) δ 8.32 (dd, *J* = 16.6, 11.3 Hz, 3H), 8.10 (dd, *J* = 14.4, 8.6 Hz, 2H), 7.80 (t, *J* = 7.3 Hz, 1H), 7.67 (t, *J* = 7.1 Hz, 1H), 7.54 (t, *J* = 7.5 Hz, 1H), 6.40 (d, *J* = 14.4 Hz, 1H), 6.08 (d, *J* = 14.0 Hz, 1H), 4.40 (q, *J* = 7.1 Hz, 2H), 2.73 (t, *J* = 5.5 Hz, 2H), 2.65 (t, *J* = 5.6 Hz, 2H), 1.95 (s, 3H), 1.94 (s, 3H), 1.89 – 1.78 (m, 2H), 1.61 (s, 6H), 1.38 (t, *J* = 7.1 Hz, 3H); Anal. calc. for $C_{36}H_{33}ClN_4O$: C, 75.4, H, 5.8, O, 2.8; found: C 75.2, H 5.7, O 2.9; HRMS (ESI MS) m/z: theor: 573.2416 found: 573.2417 ($[M+H]^+$ detected).

Synthesis of 2-(4-((*E*)-2-((*E*)-2-chloro-3-((*E*)-2-(1,1,3-trimethyl-1,3-dihydro-2*H*-benzo[*e*]indol-2-ylidene)ethylidene)cyclohex-1-en-1-yl)vinyl)-3-cyano-5,5-dimethylfuran-2(5*H*)-ylidene)malononitrile (Cb)

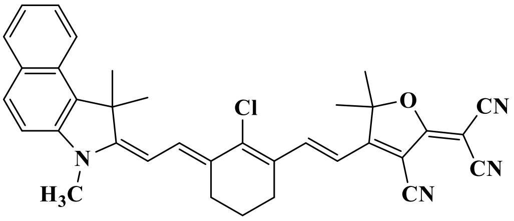

Chemical Formula: $C_{35}H_{31}ClN_4O$
Molecular Weight: 559.1100

2-(4-((*E*)-2-((*E*)-2-Chloro-3-(ethoxymethylene)cyclohex-1-en-1-yl)vinyl)-3-cyano-5,5-dimethylfuran-2(5*H*)-ylidene)malononitrile (0.5 g, 1.31 mmol, 1 equiv, *M* = 381.86 g/mol) and 1,1,2,3-tetramethyl-1*H*-benzo[*e*]indol-3-ium iodide (0.55 g, 1.57 mmol, 1.2 equiv, *M* = 351.23 g/mol) were dissolved in ethanol (50 mL), and anhydrous pyridine (0.16 g, 0.16 mL, 1.96 mmol, 1.5 equiv, *M* = 79.10 g/mol, ρ = 0.978) was added. The reaction mixture was stirred for 18 h at 80 °C. The solvent was evaporated under reduced pressure, the residue was dissolved in 20 mL of dichloromethane and washed with distilled water (2 × 20 mL). The organic layer was dried over anhydrous magnesium sulfate, filtered, and concentrated. The crude solid was purified by flash chromatography on silica gel (manual chromatography using air pressure to speed up the flow, silica gel high purity grade, pore size 60 Å, 230–400 mesh particle size, 40–63 µm particle size, for flash chromatography, Sigma Aldrich) using dichloromethane as eluent to afford the product as a green solid (688 mg, 94% yield). ^{1}H NMR (400 MHz, DMSO-d_6) δ 8.39 – 8.24 (m, 3H), 8.10 (dd, *J* = 15.1, 8.4 Hz, 2H), 7.80 (d, *J* = 8.9 Hz, 1H), 7.67 (t, *J* = 7.7 Hz, 1H), 7.54 (t, *J* = 7.4 Hz, 1H), 6.40 (d, *J* = 14.6 Hz, 1H), 6.06 (d, *J* = 14.0 Hz, 1H), 3.81 (d, *J* = 25.3 Hz, 3H), 2.72 (s, 2H), 2.65 (s, 2H), 1.94 (s, 6H), 1.88 – 1.80 (m, 2H), 1.61 (s, 6H); Anal. calc. for $C_{35}H_{31}ClN_4O$: C, 75.2, H, 5.6, O, 2.9; found: C 75.4, H 5.8, O 2.9; HRMS (ESI MS) m/z: theor: 559.2259 found: 559.2261 ($[M+H]^+$ detected).

Synthesis of sodium ((*Z*)-4-((*E*)-2-(2-chloro-3-((*E*)-2-(4-cyano-5-(dicyanomethylene)-2,2-dimethyl-2,5-dihydrofuran-3-yl)vinyl)cyclohex-2-en-1-ylidene)ethylidene)-3-cyano-5,5-dimethyl-4,5-dihydrofuran-2-yl)dicyanomethanide (CNa)

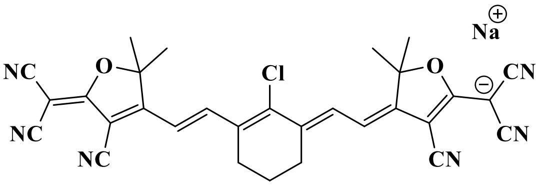

Chemical Formula: $C_{30}H_{22}ClN_6NaO_2$
Molecular Weight: 556.9858

(*Z*)-2-Chloro-3-(hydroxymethylene)cyclohex-1-ene-1-carbaldehyde (1.73 g, 10.0 mmol, M = 172.61 g/mol) and 2-(3-cyano-4,5,5-trimethylfuran-2(5*H*)-ylidene)malononitrile (4.0 g, 20.0 mmol, M = 199.21 g/mol), 500 mg CH_3COONa, and 10 mL acetic anhydride were added to a 50 mL round-bottomed flask. The mixture was heated to 70 °C for reaction 1 h under the nitrogen atmosphere. The mixture cooled down to 25 °C and with saturated $NaHCO_3$ neutralization and the precipitate filtered to afford the crude products and washed with large amount of dichloromethane until the filtrate became colorless (from orange, 4.57 g, 82% yield). ^{1}H NMR (300 MHz, Acetone) δ 8.29 (d, J = 14.3 Hz, 2H), 6.13 (d, J = 14.2 Hz, 2H), 2.63 (t, J = 6.1 Hz, 4H), 1.91 – 1.80 (m, 2H), 1.69 (s, J = 9.1 Hz, 12H); Anal. calc. for $C_{30}H_{22}ClN_6NaO_2$: C, 64.7, H, 4.0, O, 5.7; found: C 64.9, H 4.2, O 5.5; HRMS (ESI MS) m/z: theor: 533.1498 found: 533.1495 (M^- detected).

Synthesis of 2-((*E*)-2-((*E*)-2-chloro-3-((*Z*)-2-(3-hexylbenzo[*d*]thiazol-2(3*H*)-ylidene) ethylidene)cyclohex-1-en-1-yl)vinyl)-3-hexylbenzo[*d*]thiazol-3-ium tetraphenylborate (CBPh1)

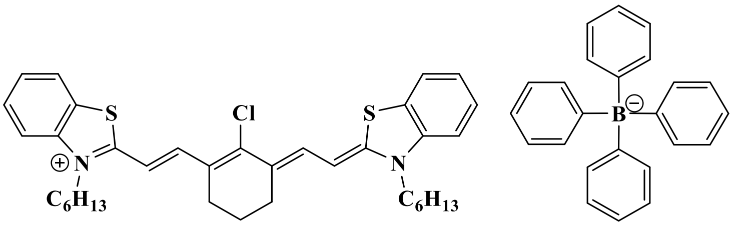

Chemical Formula: $C_{60}H_{64}BClN_2S_2$
Molecular Weight: 923.5660

2-((*E*)-2-((*E*)-2-Chloro-3-((*Z*)-2-(3-hexylbenzo[*d*]thiazol-2(3*H*)-ylidene)ethylidene)cyclohex-1-en-1-yl)vinyl)-3-hexylbenzo[*d*]thiazol-3-ium iodide (0.73 g, 1 mmol, *M* = 731.23 g/mol) was suspended in acetonitrile (10 mL) and sodium tetraphenylborate (0.35 g, 1 mmol, *M* = 342.22 g/mol) dissolved in water (5 mL) was added. The solution was stirred at room temperature overnight. Acetonitrile was evaporated under reduced pressure and a brown solid was filtered, washed several times with pentane and dried under vacuum (785 mg, 85% yield). ^{1}H NMR (400 MHz, $CDCl_3$) δ 7.93 (d, *J* = 13.4 Hz, 2H), 7.57 (d, *J* = 7.6 Hz, 2H), 7.47 – 7.38 (m, 10H), 7.31 (t, *J* = 7.6 Hz, 2H), 7.08 (d, *J* = 8.3 Hz, 2H), 6.99 (t, *J* = 7.3 Hz, 8H), 6.84 (t, *J* = 7.1 Hz, 4H), 5.98 (d, *J* = 13.4 Hz, 2H), 3.79 (t, *J* = 7.3 Hz, 4H), 2.52 (t, *J* = 6.0 Hz, 4H), 1.95 – 1.85 (m, 2H), 1.69 (m, 4H), 1.55 (m, 4H), 1.32 (m, 8H), 0.90 (t, *J* = 6.4 Hz, 6H); Anal. calc. for $C_{60}H_{64}BClN_2S_2$: C, 78.0, H, 7.0, N, 3.0; found: C 77.8, H 7.2, N 3.1; HRMS (ESI MS) m/z: theor: 603.2629 found: 603.2630 (M^+ detected).

Synthesis of 2-((*E*)-2-((*E*)-2-chloro-3-((*E*)-2-(1,1,3-trimethyl-1,3-dihydro-2*H*-benzo[*e*]indol-2-ylidene)ethylidene)cyclohex-1-en-1-yl)vinyl)-1,1,3-trimethyl-1*H*-benzo[*e*]indol-3-ium tetraphenylborate (CBPh2)

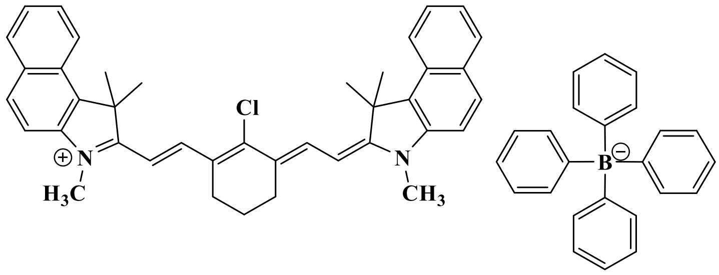

Chemical Formula: $C_{64}H_{60}BClN_2$
Molecular Weight: 903.4580

2-((*E*)-2-((*E*)-2-chloro-3-((*E*)-2-(1,1,3-trimethyl-1,3-dihydro-2*H*-benzo[*e*]indol-2-ylidene) ethylidene)cyclohex-1-en-1-yl)vinyl)-1,1,3-trimethyl-1*H*-benzo[*e*]indol-3-ium iodide (0.71 g, 1 mmol, *M* = 711.13 g/mol) was suspended in acetonitrile (10 mL) and sodium tetraphenylborate (0.34 g, 1 mmol, *M* = 342.22 g/mol) dissolved in water (5 mL) was added. The solution was stirred at room temperature overnight. Acetonitrile was evaporated under reduced pressure and a brown solid was filtered, washed several times with pentane and dried under vacuum (822 mg, 91% yield). ^{1}H NMR (400 MHz, $CDCl_3$) δ 8.42 (d, *J* = 14.4 Hz, 2H), 8.11 (d, *J* = 8.5 Hz, 2H), 7.94 (d, *J* = 8.2 Hz, 2H), 7.90 (d, *J* = 8.8 Hz, 2H), 7.64 – 7.57 (m, 2H), 7.50 (m, 10H), 7.17 (d, *J* = 8.8 Hz, 2H), 7.01 (t, *J* = 7.3 Hz, 8H), 6.83 (t, *J* = 7.2 Hz, 4H), 5.91 (d, *J* = 14.2 Hz, 2H), 3.17 (s, 6H), 2.60 (t, *J* = 5.7 Hz, 4H), 1.99 (s, 12H), 1.92 (m, 2H); Anal. calc. for $C_{64}H_{60}BClN_2$: C, 85.1, H, 6.7, N, 3.1; found: C 84.9, H 6.6, N 3.2; HRMS (ESI MS) m/z: theor: 583.2875 found: 583.2879 (M^+ detected).

Synthesis of 2-((*E*)-2-((*E*)-2-chloro-3-((*E*)-2-(1-hexylbenzo[*cd*]indol-2(1*H*)-ylidene) ethylidene)cyclohex-1-en-1-yl)vinyl)-1-hexylbenzo[*cd*]indol-1-ium tetraphenylborate (CBPh3)

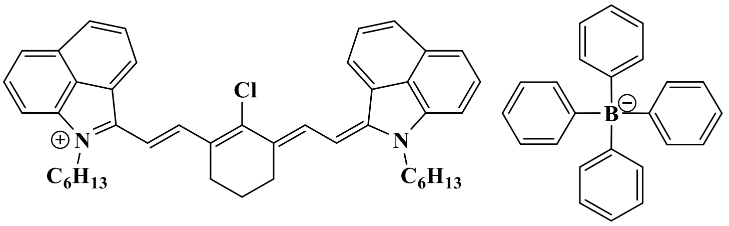

Chemical Formula: $C_{68}H_{68}BClN_2$
Molecular Weight: 959.5660

2-((*E*)-2-((*E*)-2-chloro-3-((*E*)-2-(1-hexylbenzo[*cd*]indol-2(1*H*)-ylidene)ethylidene)cyclohex-1-en-1-yl)vinyl)-1-hexylbenzo[*cd*]indol-1-ium iodide (0.77 g, 1 mmol, *M* = 767.24 g/mol) was suspended in acetonitrile (10 mL) and sodium tetraphenylborate (0.35 g, 1 mmol, *M* = 342.22 g/mol) dissolved in water (5 mL) was added. The solution was stirred at room temperature overnight. Acetonitrile was evaporated under reduced pressure and a brown solid was filtered, washed several times with pentane and dried under vacuum (844 mg, 88% yield). ^{1}H NMR (400 MHz, $CDCl_3$) δ 8.61 (d, *J* = 12.0 Hz, 2H), 8.25 (d, *J* = 5.9 Hz, 2H), 8.01 (d, *J* = 7.7 Hz, 2H), 7.88 (t, *J* = 7.7 Hz, 2H), 7.54 (d, *J* = 8.2 Hz, 2H), 7.48 (t, *J* = 7.7 Hz, 2H), 7.14 (d, *J* = 6.7 Hz, 2H), 7.40 (m, 8H), 6.94 (t, J = 7.2 Hz, 8H), 6.79 (t, J = 7.0 Hz, 4H), 6.55 (d, *J* = 14.0 Hz, 2H), 3.94 (t, *J* = 7.1 Hz, 4H), 2.88 (m, 4H), 2.08 (m, 2H), 1.81 (dt, *J* = 15.0, 7.5 Hz, 4H), 1.45 – 1.39 (m, 4H), 1.36 – 1.25 (m, 8H), 0.88 (t, *J* = 7.0 Hz, 6H); Anal. calc. for $C_{68}H_{68}BClN_2$: C, 85.1, H, 7.1, N, 2.9; found: C 85.3, H 7.2, N 3.1; HRMS (ESI MS) m/z: theor: 639.3501 found: 639.3505 (M^+ detected).

Synthesis of 2-((*E*)-2-((*E*)-2-chloro-3-(2-((*E*)-1-hexyl-3,3-dimethylindolin-2-ylidene) ethylidene)cyclohex-1-en-1-yl)vinyl)-1-hexyl-3,3-dimethyl-3*H*-indol-1-ium tetraphenylborate (CBPh4)

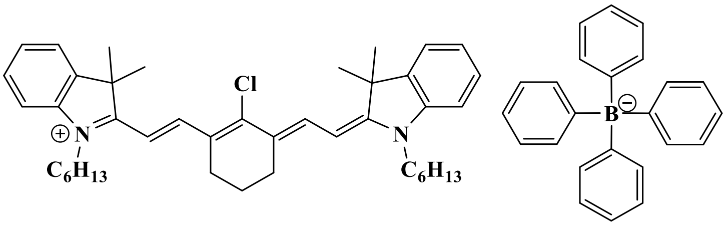

Chemical Formula: $C_{66}H_{76}BClN_2$
Molecular Weight: 943.6080

2-((*E*)-2-((*E*)-2-Chloro-3-(2-((*E*)-1-hexyl-3,3-dimethylindolin-2-ylidene)ethylidene)cyclohex-1-en-1-yl)vinyl)-1-hexyl-3,3-dimethyl-3*H*-indol-1-ium iodide (0.75 g, 1 mmol, *M* = 751.28 g/mol) was suspended in acetonitrile (10 mL) and sodium tetraphenylborate (0.35 g, 1 mmol, *M* = 342.22 g/mol) dissolved in water (5 mL) was added. The solution was stirred at room temperature overnight. Acetonitrile was evaporated under reduced pressure and the resulting brown solid was filtered off, washed several times with pentane and dried under vacuum (896 mg, 95% yield). ^{1}H NMR (400 MHz, $CDCl_3$) δ 0.89 (t, 6H, *J* = 6.9 Hz), 1.30-1.38 (m, 8H), 1.39-1.49 (m, 4H), 1.72 (s, 12H), 1.85 (qt, 4H, *J* = 6.7 Hz), 2.75 (t, 4H, *J* = 7.1 Hz), 4.20 (t, 4H, J = 7.2 Hz), 6.23-6.29 (m, 2H), 6.88 (t, *J* = 7.1 Hz, 4H), 7.02 (t, *J* = 7.4 Hz, 8H), 7.15 (d, 2H, *J* = 7.9 Hz), 7.23-7.27 (m, 2H), 7.37-7.42 (m, 4H), 7.46 (m, br, 8H), 8.20-8.35 (m, 2H); Anal. calc. for $C_{66}H_{76}BClN_2$: C, 84.0, H, 8.1, N, 3.0; found: C 83.8, H 8.2, N 3.2; HRMS (ESI MS) m/z: theor: 623.4127 found: 623.4124 (M^+ detected).

Synthesis of 2-((*E*)-2-((*E*)-2-Chloro-3-((*Z*)-2-(3-hexylbenzo[*d*]thiazol-2(3*H*)-ylidene) ethylidene)cyclohex-1-en-1-yl)vinyl)-3-hexylbenzo[*d*]thiazol-3-ium iodide (CI1)

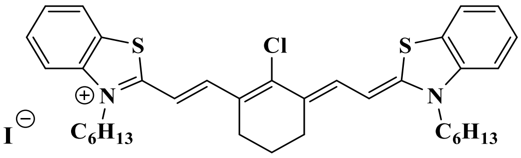

Chemical Formula: $C_{36}H_{44}ClIN_2S_2$
Molecular Weight: 731.2365

2-Chloro-3-(hydroxymethylene)cyclohex-1-ene-1-carbaldehyde (0.5 g, 2.9 mmol, 1 equiv, *M* = 172.61 g/mol) and 3-hexyl-2-methylbenzo[*d*]thiazol-3-ium iodide (2.10 g, 5.81 mmol, 2 equiv, *M* = 361.28 g/mol) were dissolved in butan-1-ol/toluene 7:3 (300 mL) and the solution was refluxed at 160 °C overnight with a Dean–Stark condenser. After cooling, the solvent was evaporated, and the resulting green solid was purified by column chromatography (SiO_2), using a gradient of solvent from DCM to acetone (1.74 g, 82% yield). ^1H NMR (400 MHz, $CDCl_3$) δ 0.88 (t, 6H, *J* = 7.0 Hz), 1.25-1.37 (m, 8H), 1.46 (qt, 4H, *J* = 7.0 Hz), 1.83 (qt, 4H, *J* = 7.3 Hz), 1.94 (qt, 2H, *J* = 5.9 Hz), 2.67 (t, 4H, *J* = 7.4 Hz), 4.38 (t, 4H, *J* = 7.4 Hz), 6.30 (d, 2H, *J* = 13.4 Hz), 7.29 (td, 2H, *J* = 8.0 Hz, *J* = 0.8 Hz), 7.37 (d, 2H, *J* = 8.1 Hz), 7.46 (td, 2H, *J* = 8.3 Hz, *J* = 1.1 Hz), 7.73 (d, 2H, *J* = 7.2 Hz), 7.86 (d, 2H, *J* = 13.4 Hz); ^{13}C NMR (100 MHz, $CDCl_3$) δ 14.0, 20.9, 22.4, 26.5, 27.3, 27.8, 31.4, 99.5, 112.9, 122.8, 125.2, 125.6, 125.8, 128.2, 141.6, 142.0, 147.0, 163.1; HRMS (ESI MS) m/z: theor: 603.2629 found: 603.2628 (M^+ detected).

Synthesis of 2-((E)-2-((E)-2-chloro-3-((E)-2-(1,1,3-trimethyl-1,3-dihydro-2H-benzo[e]indol-2-ylidene)ethylidene)cyclohex-1-en-1-yl)vinyl)-1,1,3-trimethyl-1H-benzo[e]indol-3-ium iodide (CI2)

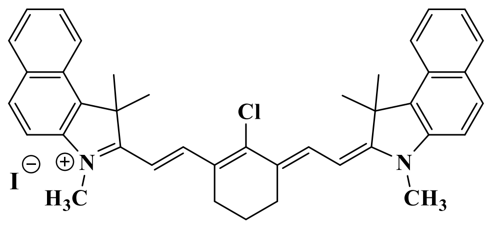

Chemical Formula: $C_{40}H_{40}ClIN_2$
Molecular Weight: 711.1285

2-Chloro-3-(hydroxymethylene)cyclohex-1-ene-1-carbaldehyde (0.5 g, 2.9 mmol, 1 equiv, *M* = 172.61 g/mol) and 1,1,2,3-tetramethyl-1*H*-benzo[*e*]indol-3-ium iodide (2.04 g, 5.81 mmol, 2 equiv, *M* = 351.22 g/mol) were dissolved in butan-1-ol/toluene (7:3) (300 mL) and the solution was refluxed at 160 °C overnight with a Dean-Stark condenser. After cooling, the solvent was evaporated, and the resulting green solid was purified by column chromatography (SiO_2), using a gradient of solvent from DCM to acetone (1.75 g, 85% yield). ^{1}H NMR (400 MHz, $CDCl_3$) δ 8.46 (d, *J* = 14.0 Hz, 2H), 8.13 (d, *J* = 8.6 Hz, 2H), 7.96 (dd, *J* = 8.5, 2.6 Hz, 4H), 7.62 (t, *J* = 7.1 Hz, 2H), 7.48 (t, *J* = 8.2 Hz, 4H), 6.34 – 6.25 (m, 2H), 3.89 (s, 6H), 2.81 (t, *J* = 5.7 Hz, 2H), 2.04 (s, 12H), 1.56 (d, *J* = 13.8 Hz, 4H); Anal. calc. for $C_{40}H_{40}ClIN_2$: C, 67.6, H, 5.7, N, 3.9; found: C 67.8, H 5.8, N 3.7; HRMS (ESI MS) m/z: theor: 583.2875 found: 583.2876 (M^+ detected).

Synthesis of 2-((*E*)-2-((*E*)-2-chloro-3-((*E*)-2-(1-hexylbenzo[*cd*]indol-2(1*H*)-ylidene) ethylidene)cyclohex-1-en-1-yl)vinyl)-1-hexylbenzo[*cd*]indol-1-ium iodide (CI3)

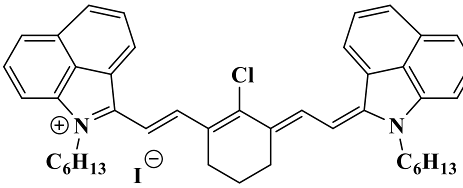

Chemical Formula: $C_{44}H_{48}ClIN_2$
Molecular Weight: 767.2365

To a solution of 1-hexyl-2-methylbenzo[*cd*]indol-1-ium iodide (3.79 g, 10.0 mmol, *M* = 379.29 g/mol) and 2-chloro-3-(hydroxymethylene)cyclohex-1-ene-1-carbaldehyde (0.86 g, 5.0 mmol, *M* = 172.61 g/mol) in 10 mL of acetic acid, 5.0 mL of triethylamine and 5.0 mL of acetic anhydride were added and reacted at 60 °C for 30 min. Then, the mixture was cooled, and 100 mL of ethyl acetate was added. The black precipitate was filtered and recrystallized in EtOH (2.54 g, 66% yield) ^{1}H NMR (400 MHz, $CDCl_3$) δ 8.61 (d, *J* = 12.0 Hz, 2H), 8.25 (d, *J* = 5.9 Hz, 2H), 8.01 (d, *J* = 7.7 Hz, 2H), 7.88 (t, *J* = 7.7 Hz, 2H), 7.54 (d, *J* = 8.2 Hz, 2H), 7.48 (t, *J* = 7.7 Hz, 2H), 7.14 (d, *J* = 6.7 Hz, 2H), 6.55 (d, *J* = 14.0 Hz, 2H), 4.13 (t, *J* = 7.1 Hz, 4H), 2.88 (m, 4H), 2.08 (m, 2H), 1.81 (dt, *J* = 15.0, 7.5 Hz, 4H), 1.45 – 1.39 (m, 4H), 1.36 – 1.25 (m, 8H), 0.88 (t, *J* = 7.0 Hz, 6H); Anal. calc. for $C_{44}H_{48}ClIN_2$: C, 68.9, H, 6.3, N, 3.6; found: C 68.8, H 6.2, N 3.4; HRMS (ESI MS) m/z: theor: 639.3501 found: 639.3502 (M^+ detected).

Synthesis of 2-((*E*)-2-((*E*)-2-chloro-3-(2-((*E*)-1-hexyl-3,3-dimethylindolin-2-ylidene) ethylidene)cyclohex-1-en-1-yl)vinyl)-1-hexyl-3,3-dimethyl-3*H*-indol-1-ium iodide (CI4)

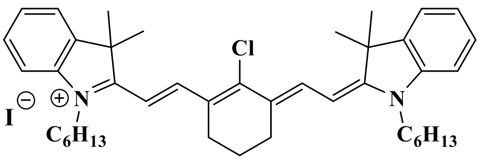

Chemical Formula: $C_{42}H_{56}ClIN_2$
Molecular Weight: 751.2785

2-Chloro-3-(hydroxymethylene)cyclohex-1-ene-1-carbaldehyde (0.5 g, 2.9 mmol, 1 equiv, *M* = 172.61 g/mol) and 1-hexyl-2,3,3-trimethyl-3*H*-indol-1-ium iodide (2.16 g, 5.81 mmol, 2 equiv, *M* = 371.31 g/mol) were dissolved in butan-1-ol/toluene 7:3 (300 mL) and the solution was refluxed at 160 °C overnight with a Dean–Stark condenser. After cooling, the solvent was evaporated, and the resulting green solid was purified by column chromatography (SiO_2), using a gradient of solvent from DCM to acetone (1.98 g, 91% yield). ^{1}H NMR (400 MHz, $CDCl_3$) δ 0.89 (t, 6H, J = 6.9 Hz), 1.30-1.38 (m, 8H), 1.39-1.49 (m, 4H), 1.72 (s, 12H), 1.85 (qt, 4H, J = 6.7 Hz), 2.75 (t, 4H, J = 7.1 Hz), 4.20 (t, 4H, J = 7.2 Hz), 6.23-6.29 (m, 2H), 7.15 (d, 2H, J = 7.9 Hz), 7.23-7.27 (m, 2H), 7.37-7.42 (m, 4H), 8.20-8.35 (m, 2H); ^{13}C NMR (100 MHz, $CDCl_3$) δ 14.0, 20.7, 22.4, 26.6, 26.7, 27.4, 28.0, 28.1, 31.4, 45.1, 49.3, 49.4, 101.4, 102.2, 110.9, 122.3, 125.3, 127.4, 128.8, 133.4, 141.1, 141.3, 142.3, 144.2, 150.4, 154.0, 172.3; HRMS (ESI MS) m/z: theor: 623.4127 found: 623.4130 (M^+ detected).

Synthesis of 2-((*E*)-2-((*E*)-1-benzyl-4-chloro-5-(2-((*E*)-1-hexyl-3,3-dimethylindolin-2-ylidene) ethylidene)-1,2,5,6-tetrahydropyridin-3-yl)vinyl)-1-hexyl-3,3-dimethyl-3*H*-indol-1-ium iodide (CI6)

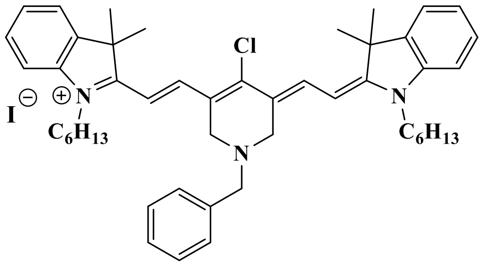

Chemical Formula: $C_{48}H_{61}ClIN_3$
Molecular Weight: 842.3915

1-Benzyl-4-chloro-5-(hydroxymethylene)-1,2,5,6-tetrahydropyridine-3-carbaldehyde (0.76 g, 2.9 mmol, 1 equiv, M = 263.72 g/mol) and 3-ethyl-1,1,2-trimethyl-1*H*-benzo[*e*]indol-3-ium iodide (2.12 g, 5.8 mmol, 2 equiv, M = 365.26 g/mol) were dissolved in butan-1-ol/toluene 7:3 (300 mL) and the solution was refluxed at 160 °C overnight with a Dean–Stark condenser. After cooling, the solvent was evaporated. Dissolution in a minimum of acetone (5 mL) followed by pentane precipitated a brown solid that was filtered off, washed several times with pentane and dried under vacuum (2.10 g, 86% yield). ^{1}H NMR (400 MHz, $CDCl_3$) δ 8.19 (d, J = 14.4 Hz, 2H), 7.56 (d, J = 27.5 Hz, 2H), 7.46 – 7.22 (m, 9H), 7.14 (d, J = 7.9 Hz, 2H), 5.99 (d, J = 14.4 Hz, 2H), 4.13 (t, J = 7.3 Hz, 4H), 3.89 (s, 2H), 1.82 – 1.64 (m, 16H), 1.45 – 1.23 (m, 16H), 0.90 (t, J = 7.0 Hz, 6H); Anal. calc. for $C_{48}H_{61}ClIN_3$: C, 68.4, H, 7.3, N, 5.0; found: C 68.5, H 7.1, N 5.2; HRMS (ESI MS) m/z: theor: 714.4549 found: 714.4546 (M^+ detected).

Synthesis of 2-((*E*)-2-((*E*)-5-(*tert*-butyl)-2-chloro-3-((*E*)-2-(1,1,3-trimethyl-1,3-dihydro-2*H*-benzo[*e*]indol-2-ylidene)ethylidene)cyclohex-1-en-1-yl)vinyl)-1,1,3-trimethyl-1*H*-benzo[*e*] indol-3-ium iodide (CI7)

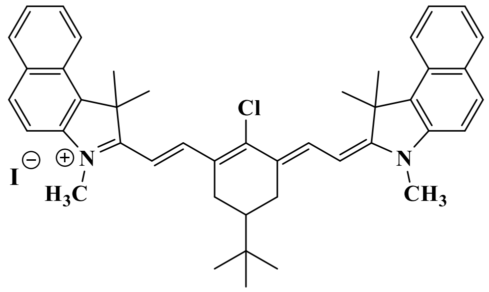

Chemical Formula: $C_{44}H_{48}ClIN_2$
Molecular Weight: 767.2365

5-(*Tert*-butyl)-2-chloro-3-(hydroxymethylene)cyclohex-1-ene-1-carbaldehyde (0.66 g, 2.9 mmol, 1 equiv, *M* = 228.72 g/mol) and 1,1,2,3-tetramethyl-1*H*-benzo[*e*]indol-3-ium iodide (2.04 g, 5.81 mmol, 2 equiv, *M* = 351.22 g/mol) were dissolved in butan-1-ol/toluene 7:3 (300 mL) and the solution was refluxed at 160 °C overnight with a Dean–Stark condenser. After cooling, the solvent was evaporated, and the resulting green solid was purified by column chromatography (SiO_2), using a gradient of solvent from DCM to acetone (1.96 g, 88% yield). ^{1}H NMR (400 MHz, DMSO-d_6) δ 8.47 (d, *J* = 14.2 Hz, 2H), 8.15 – 8.08 (m, 2H), 7.96 (t, *J* = 7.9 Hz, 4H), 7.62 (t, *J* = 7.7 Hz, 2H), 7.50 (dd, *J* = 17.0, 8.1 Hz, 4H), 6.19 (d, *J* = 14.2 Hz, 2H), 3.88 (s, 6H), 3.65 (t, *J* = 6.6 Hz, 1H), 3.00 – 2.89 (m, 2H), 2.31 (dd, *J* = 25.2, 11.2 Hz, 2H), 2.04 (s, 12H), 1.13 (s, 9H); Anal. calc. for $C_{44}H_{48}ClIN_2$: C, 68.9, H, 6.3, N, 3.6; found: C 68.7, H 6.1, N 3.7; HRMS (ESI MS) m/z: theor: 639.3501 found: 639.3504 (M^+ detected).

Synthesis of 2-((*E*)-2-((*E*)-5-(*tert*-butyl)-2-chloro-3-(2-((*E*)-1-hexyl-3,3-dimethylindolin-2-ylidene)ethylidene)cyclohex-1-en-1-yl)vinyl)-1-hexyl-3,3-dimethyl-3*H*-indol-1-ium iodide (CI8)

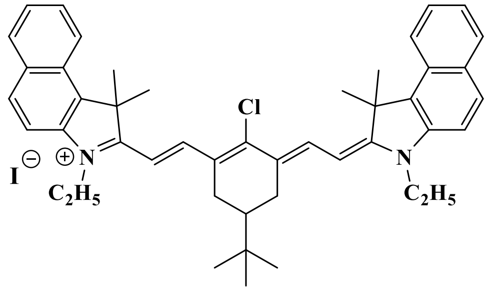

Chemical Formula: $C_{46}H_{52}ClIN_2$
Molecular Weight: 795.2905

5-(*Tert*-butyl)-2-chloro-3-(hydroxymethylene)cyclohex-1-ene-1-carbaldehyde (0.66 g, 2.9 mmol, 1 equiv, *M* = 228.72 g/mol) and 3-ethyl-1,1,2-trimethyl-1*H*-benzo[*e*]indol-3-ium iodide (2.12 g, 5.81 mmol, 2 equiv, *M* = 365.26 g/mol) were dissolved in butan-1-ol/toluene 7:3 (300 mL) and the solution was refluxed at 160 °C overnight with a Dean–Stark condenser. After cooling, the solvent was evaporated, and the resulting green solid was purified by column chromatography (SiO_2), using a gradient of solvent from DCM to acetone (2.08 g, 90% yield). ^1H NMR (400 MHz, $CDCl_3$) δ 8.46 (t, *J* = 12.5 Hz, 2H), 8.13 (d, *J* = 8.7 Hz, 2H), 8.04 – 7.92 (m, 4H), 7.62 (t, *J* = 7.1 Hz, 2H), 7.50 (dd, *J* = 12.1, 5.5 Hz, 4H), 6.20 (d, *J* = 14.2 Hz, 2H), 4.50 – 4.29 (m, 4H), 2.93 (d, *J* = 12.0 Hz, 2H), 2.39 – 2.22 (m, 2H), 2.03 (s, 12H), 1.54 (t, *J* = 7.2 Hz, 6H), 1.15 – 1.06 (m, 10H); Anal. calc. for $C_{46}H_{52}ClIN_2$: C, 69.5, H, 6.6, N, 3.5; found: C 69.5, H 6.8, N 3.4; HRMS (ESI MS) m/z: theor: 667.3814 found: 667.3818 (M^+ detected). Analyses are consistent with those previously reported in the literature [5].

Synthesis of 2-((*E*)-2-((*E*)-5-(*tert*-butyl)-2-chloro-3-(2-((*E*)-1-hexyl-3,3-dimethylindolin-2-ylidene)ethylidene)cyclohex-1-en-1-yl)vinyl)-1-hexyl-3,3-dimethyl-3*H*-indol-1-ium iodide (CI9)

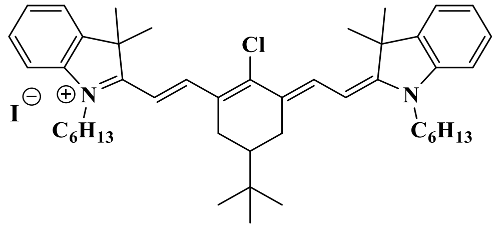

Chemical Formula: $C_{46}H_{64}ClIN_2$
Molecular Weight: 807.3865

5-(*Tert*-butyl)-2-chloro-3-(hydroxymethylene)cyclohex-1-ene-1-carbaldehyde (0.66 g, 2.9 mmol, 1 equiv, M = 228.72 g/mol) and 1-hexyl-2,3,3-trimethyl-3*H*-indol-1-ium iodide (2.16 g, 5.81 mmol, 2 equiv, M = 371.31 g/mol) were dissolved in butan-1-ol/toluene (7:3) (300 mL) and the solution was refluxed at 160 °C overnight with a Dean–Stark condenser. After cooling, the solvent was evaporated. Dissolution in a minimum of acetone (5 mL), followed by pentane precipitated a brown solid that was filtered off, washed several times with pentane and dried under vacuum (1.97 g, 84% yield). ^{1}H NMR (400 MHz, $CDCl_3$) δ 8.23 (dd, J = 36.1, 14.0 Hz, 2H), 7.34 (t, J = 8.2 Hz, 4H), 7.24 – 7.11 (m, 5H), 6.16 – 6.05 (m, 2H), 4.12 (qd, J = 14.5, 7.3 Hz, 4H), 2.81 (d, J = 15.2 Hz, 2H), 2.28 – 2.11 (m, 2H), 1.84 – 1.74 (m, 4H), 1.72 (s, 2H), 1.66 (d, J = 1.7 Hz, 9H), 1.57 (s, 2H), 1.40 (d, J = 6.8 Hz, 4H), 1.29 (t, J = 14.3 Hz, 8H), 1.03 (d, J = 3.4 Hz, 8H), 0.82 (td, J = 6.7, 2.7 Hz, 6H); Anal. calc. for $C_{46}H_{64}ClIN_2$: C, 68.4, H, 8.0, N, 3.5; found: C 68.5, H 7.8, N 3.4; HRMS (ESI MS) m/z: theor: 679.4753 found: 679.4751 (M^+ detected).

Synthesis of 1-hexyl-2-methylbenzo[cd]indol-1-ium iodide (CI10)

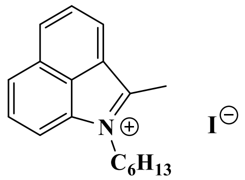

Chemical Formula: $C_{18}H_{22}IN$
Molecular Weight: 379.2855

1-Hexylbenzo[*cd*]indol-2(1*H*)-one (7.60 g, 30.0 mmol, *M* = 253.34 g/mol) was dissolved in dry THF (30 mL) and a solution of MeMgCl (13.0 mL, 3.0 *M* in THF, 1.3 equiv) was added dropwise. The reaction mixture was heated to 55 °C during 60 min. After return to room temperature, the reaction mixture was poured slowly into an ice/water mixture (150 mL) containing 12.0 mL of HCl 32%. THF was removed under reduced pressure, the aqueous mixture was filtered, and the clear filtrate added to a stirred solution of KI (9.90 g, 60.0 mmol, 2.00 eq) in water (150 mL). The red precipitate was filtered, washed with water and ethyl acetate and dried under vacuum to give a red solid (5.72 g, 15.08 mmol, 50% yield). ^{1}H NMR (400 MHz, $CDCl_3$) δ 8.96 (d, *J* = 7.3 Hz, 1H), 8.60 (d, *J* = 8.1 Hz, 1H), 8.40 (d, *J* = 7.4 Hz, 1H), 8.27 (d, *J* = 8.2 Hz, 1H), 8.03 (t, *J* = 7.7 Hz, 1H), 7.87 (t, *J* = 7.8 Hz, 1H), 4.83 (t, *J* = 7.5 Hz, 2H), 3.45 (s, 3H), 2.07 – 1.92 (m, 2H), 1.54 – 1.41 (m, 2H), 1.36 – 1.20 (m, 4H), 0.81 (t, *J* = 7.1 Hz, 3H); Anal. calc. for $C_{18}H_{22}IN$: C, 57.0, H, 5.8, N, 3.7; found: C 57.2, H 5.8, N 3.4; HRMS (ESI MS) m/z: theor: 380.0870 found: 380.0872 (M^+ detected).

Photopolymerization procedure

The efficiency of the different PISs was checked with a benchmark multifunctional monomer (pentaerythritol triacrylate, PETIA) containing acrylate functions (see Scheme 2 in the main manuscript). The stabilizers were not removed before usage. The kinetic properties of the polymerization of the photosensitive formulations (acrylate function conversion vs irradiation time) were followed by real-time Fourier transform infrared (RTFTIR) spectroscopy (on a

JASCO 6600 device) in the near-infrared region (peak at 6100–6220 cm^{-1}). Photosensitive formulations were deposited in a mold (controlled thickness of 1.4 mm) and polymerized under air upon irradiation with NIR light. The irradiation starts at t = 10 s after the first spectrum measurement. More details about the photopolymerization experiments are given in the figure captions. All experiments were carried out at room temperature and under air. Each measurement has been repeated twice, and a very good repeatability was found. A laser diode at 785 nm with an irradiance of 0.9 W/cm^2 was used as the irradiation device. For interpenetrating polymer network (IPN) synthesis with NIR light, 3,4-epoxycyclohexylmethyl-3,4-epoxycyclohexane carboxylate from Allnex was used as a difunctional epoxy monomer.

Light absorption properties of the different dyes

The UV–visible–NIR absorption spectra of the NIR dyes were acquired using a JASCO V730 spectrophotometer.

References

[1] Samanta, A.; Vendrell, M.; Das, R.; Chang, Y-T. *Chem. Commun*. **2010**, *46*, 7406.

[2] Huang, B.; Chen, W.; Kuang, Y.Q.; Liu, W.; Liu, X. J.; Tang, L. J.; Jiang, J. H. *Org. Biomol. Chem***.** **2017**, *15*, 4383.

[3] Kiyose, K.; Hanaoka, K.; Oushiki, D.; Nakamura, T.; Kajimura, M.; Suematsu, M.; Nishimatsu, H.; Yamane, T.; Terai, T.; Hirata, Y.; Nagano, T. *J. Am. Chem. Soc*. **2010**, *132*, 15846.

[4] Connell, A.; Holliman, P. J.; Davies, M. L.; Gwenin, C. D.; Weiss, S.; Pitak, M. B.; Horton, P. N.; Coles, S. J.; Cooke, G. *J. Mater. Chem. A*, **2014**, *2*, 4055.

[5] Davydenko, I.; Barlow, S.; Sharma, R.; Benis, S.; Simon, J.; Allen, T. G.; Cooper, M. W.; Khrustalev, V.; Jucov, E.V.; Castañeda, R.; Ordonez, C.; Li, Z.A.; Chi, S-H.; Jang, S-H.;

Parker, T.C.; Timofeeva, T.V.; Perry, J.W.; Jen, A. K-Y.; Hagan, D.J.; Stryland, E.V.; Marder, S.R. *J. Am. Chem. Soc.* **2016**, *138*, 10112.
